# Antibacterial Meroterpenoids, Merochlorins G–J from the Marine Bacterium *Streptomyces* sp.

**DOI:** 10.3390/md19110618

**Published:** 2021-10-30

**Authors:** Min-Ji Ryu, Prima F. Hillman, Jihye Lee, Sunghoon Hwang, Eun-Young Lee, Sun-Shin Cha, Inho Yang, Dong-Chan Oh, Sang-Jip Nam, William Fenical

**Affiliations:** 1 Department of Chemistry and Nanoscience, Ewha Womans University, Seoul 03760, Korea; ryumj624@naver.com (M.-J.R.); primafitriah@gmail.com (P.F.H.); jl3414@gmail.com (J.L.); younglee0124@naver.com (E.-Y.L.); chajung@ewha.ac.kr (S.-S.C.); 2 Laboratories of Marine New Drugs, Redone Seoul, Seoul 08594, Korea; 3 Natural Products Research Institute, College of Pharmacy, Seoul National University, Seoul 08826, Korea; sunghooi@snu.ac.kr (S.H.); dongchanoh@snu.ac.kr (D.-C.O.); 4 Department of Convergence Study on the Ocean Science and Technology, Korea Maritime and Ocean University, Busan 49112, Korea; ihyang@kmou.ac.kr; 5 Center for Marine Biotechnology and Biomedicine, Scripps Institution of Oceanography, University of California San Diego, La Jolla, CA 92093-0204, USA

**Keywords:** chlorinated meroterpenoid, merochlorins G−J, dihydronaphthalenedione precursor, ECD, DP4, antibacterial

## Abstract

Four new chlorinated meroterpenoids, merochlorins G−J (**1**−**4**), and **10**, a dihydronaphthalenedione precursor, along with known merochlorins A (**5**) and C−F (**6**−**9**), were obtained from cultivation of the bacterium strain *Streptomyces* sp. CNH-189, which was isolated from marine sediment. The planar structures of compounds **1**−**4** and **10** were elucidated by interpretation of MS, UV, and NMR spectroscopic data. The relative configurations of compounds **1**−**4** were determined via analysis of nuclear Overhauser effect (NOE) spectroscopic data, after which their absolute configurations were established by comparing the experimental electronic circular dichroism (ECD) spectra of compounds **1**−**4** to those of previously reported possible enantiomer models and DP4 calculations. Compound **3** displayed strong antibacterial activities against *Bacillus subtilis*, *Kocuria rhizophila*, and *Staphylococcus aureus*, with MIC values of 1, 2, and 2 μg/mL, respectively, whereas compound **1** exhibited weak antibacterial effects on these three strains, with a 16−32 μg/mL MIC value range.

## 1. Introduction

Marine actinomycetes are remarkable sources of novel secondary metabolites with wide chemical diversity [1]. Actinomycetales are supposed to be productive producers of a wide variety of biologically active secondary metabolites, including antibiotics, enzyme inhibitors, antivascular compounds, and antitumor compounds [2,3,4,5,6,7]. Among the many genera of marine actinomycetes, members of the genus *Streptomyces* have garnered increasing attention due to their capacity to produce novel compounds with unique structural and pharmacological properties [8,9,10]. The genus *Streptomyces* is common and bountiful in terrestrial habitats and has also been reported in marine ecosystems [11]. Lately, the investigation of chemical components for *Streptomyces* species isolated from diverse marine environments has led to the discovery of diverse bioactive secondary metabolites, such as enzyme inhibitors, anticancer agents, and antibiotics [2,12,13,14,15]. Our intensive chemical screening of the marine actinomycete strain *Streptomyces* sp. CNH-189 also resulted in the discovery of novel meroterpenoids, such as ansalactams A–D, merochlorins A–F, and meroindenon [5,6,7,8]. Ansalactams possess a distinctive spiro *γ*-lactam moiety with a modified polyketide chain, which exhibits biosynthetic plasticity between strains [16]. Ansalactams B−D exert antibacterial properties against methicillin-resistant *Staphylococcus aureus* (MRSA) with minimum inhibitory concentration (MIC) values of 31.2, 31.2, and 62.5 μg/mL, respectively [17]. Merochlorins are polyketide−terpenoid hybrid products with chlorination, which are biosynthesized from tetrahydroxynaphthalene (THN) linked to a modified C_15_ isoprene unit [18]. Merochlorins A and B possess polycyclic ring systems derived from a C_15_ isoprene unit, such as a bicyclo[3.2.1]octanone or a bicyclo[6-5-5]-fused ring system, where merochlorins C and D are formed in a linear C_15_ isoprene chain. The chemical structures of merochlorins E and F are closely related to those of merochlorins C and D, where merochlorins E and F share the same planar structure with different stereochemistry of their *gem*-dimethylmethylcyclohexane ring system. Merochlorin A reportedly achieves MIC values of 1-4 μg/mL against MRSA, whereas merochlorins E and F exhibit stronger antibacterial activity against pathogenic Gram-positive strains, with MIC values of 1−2 μg/mL. The observation of this activity suggested that the *gem*-dimethylmethylcyclohexane ring in merochlorins E and F plays an important role in the antibacterial activity [18,19]. Therefore, strain CNH-189 is a promising source for the discovery and isolation of novel antibacterial meroterpenoids.

**Figure 1:**
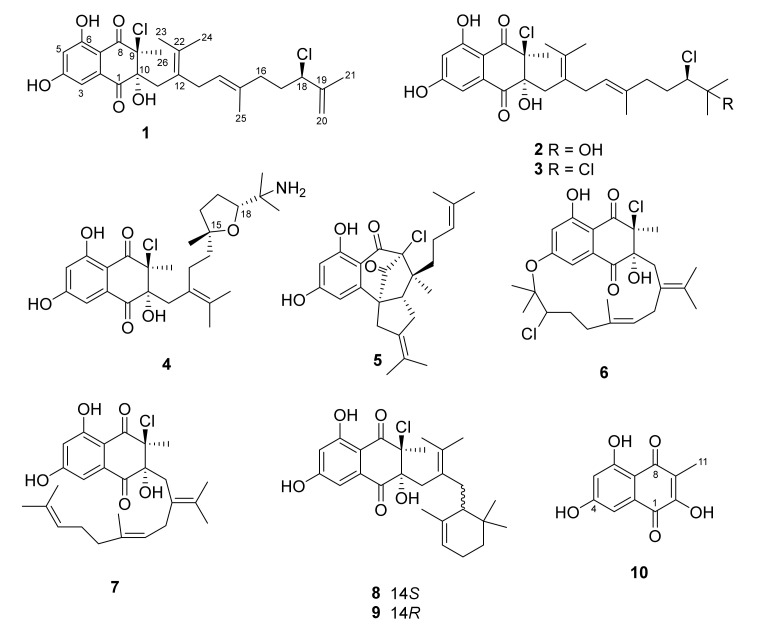
Structures of merochlorins G–J (**1**–**4**), A (**5**), C–F (**6**–**9**), and 2,5,7-trihydroxy-3-methylnaphthalene-1,4-dione (**10**).

Continuous efforts to discover novel compounds from strain CNH-189 resulted in the isolation of merochlorins G–J (**1**–**4**) and **10** from crude extracts of this bacterium, along with other known compounds, such as merochlorins A (**5**) and C–F (**6**–**9**) (Figure 1). Analyses of LC/MS, the UV profile, and characteristic MS isotopic patterns for chlorine atoms were then conducted to characterize these compounds. Here, we describe the isolation and structural characterization of merochlorins G–J (**1**–**4**) and **10**, as well as their antibacterial activity against Gram-positive bacteria.

## 2. Results and Discussion

Merochlorin G (**1**) was isolated as a white powder, and its molecular formula was determined to be C_26_H_32_^35^Cl_2_O_5_ based on HRESIMS spectral data analysis (a pseudomolecular ion peak at *m/z* 517.1517 [M + Na]^+^, calcd for C_26_H_32_^35^Cl_2_O_5_Na, 517.1524) and interpretation of ^13^C NMR data. The ^1^H NMR spectrum of **1** exhibited two aromatic protons at *δ*_H_ 7.01 and 6.71; one olefinic proton at *δ*_H_ 4.87; 10 methylenes at *δ*_H_ 4.99, 4.90, 2.84, 2.61, 2.45, 2.37, 2.05, 1.95, 1.92, and 1.87; five methyl singlets at *δ*_H_ 1.94, 1.82, 1.58, 1.51, and 1.20; and three exchangeable protons at *δ*_H_ 11.70, 5.77, and 4.08. The ^13^C NMR and HSQC spectra displayed two carbonyls at *δ*_C_ 196.1 and 194.8; 10 quaternary carbons at *δ*_C_ 165.3, 162.9, 144.3, 134.7, 134.0, 131.6, 125.1, 109.9, 84.0, and 74.5; four methine carbons at *δ*_C_ 123.5, 108.9, 107.4, and 66.4; five methylene carbons at *δ*_C_ 114.2, 38.6, 36.6, 34.8, and 31.3; and five methyl singlets at *δ*_C_ 20.9, 20.6, 18.1, 17.0, and 15.9. 

Analysis of the 2D NMR spectroscopic data of **1** allowed for the construction of two distinct fragments. Analysis of a ^1^H−^1^H coupling constant and HMBC correlations enabled the construction of the first fragment of **1**. The *meta*-coupled aromatic protons at *δ*_H_ 7.01 (H-3, 1H, d, *J* = 2.0 Hz) and 6.71 (H-5, 1H, d, *J* = 2.0 Hz), along with the observation of HMBC correlations from H-3 to C-2 (*δ*_C_ 134.7), C-4 (*δ*_C_ 162.9), C-5 (*δ*_C_ 108.9), and C-7 (*δ*_C_ 109.9) and from H-5 to C-3 (*δ*_C_ 107.4), C-4, C-6 (*δ*_C_ 165.3), and C-7, indicated the presence of a 1,2,3,5-tetra-substituted benzene moiety. Carbon chemical shifts at *δ*_C_ 162.9 and 165.3 suggested the substitutions of two aromatic hydroxy groups at C-4 and C-6, respectively. Further, HMBC correlations from the aromatic proton H-3 to C-1 (*δ*_C_ 196.1), from the methyl singlet at 1.51 (H-26, 3H, s) to C-8 (*δ*_C_ 194.8), C-9 (*δ*_C_ 74.5), and C-10 (*δ*_C_ 84.0), and from the exchangeable proton at 10-OH (*δ*_H_ 4.08, 1H, s) to C-9, C-10, and C-11 supported the presence of a dihydronaphthalenedione moiety (Table 1). 

The second fragment, the C_15_ rearranged sesquiterpene moiety, was assigned via COSY crosspeak analysis and HMBC correlations. The two methyl singlets H_3_-23 (*δ*_H_ 1.20, 3H, s) and H_3_-24 (*δ*_H_ 1.58, 3H, s) were attached at the same carbon C-22, which was further supported by the observation of the HMBC correlations between two methyl singlets (H_3_-23 and H_3_-24) and C-12 (*δ*_C_ 131.6) and C-22 (*δ*_C_ 125.1). Furthermore, long-range HMBC correlations from the methylene protons H-11 (*δ*_H_ 2.61, d, *J* = 14.0 Hz, *δ*_H_ 2.37, d *J* = 14.0 Hz) to C-12 (*δ*_C_ 131.6) and C-22 (*δ*_C_ 125.1) enabled the identification of an isoprene unit. The COSY crosspeaks between H-13 (*δ*_H_ 2.84, 1H, dd, *J* = 15.2, 6.7 Hz, *δ*_H_ 2.45, 1H, dd, *J* = 15.2, 6.7 Hz) and H-14 (*δ*_H_ 4.87, 1H, t, *J* = 7.0 Hz) and HMBC correlations from a methyl singlet H_3_-25 (*δ*_H_ 1.94, 3H, s) to C-14 (*δ*_C_ 123.5), C-15 (*δ*_C_ 134.0), and C-16 (*δ*_C_ 36.6) allowed for the isolation of another isoprene unit. The 25*E* geometry of the trisubstituted double bond was assigned based on the carbon chemical shift of 25-Me (*δ*_C_ 18.1) [10]. The last isoprene unit in the C_15_ rearranged sesquiterpene side chain was established by the COSY crosspeaks for the connectivity of H-17 (*δ*_H_ 1.92, 1H, m, *δ*_H_ 1.87, 1H, m) and H-18 (*δ*_H_ 4.33, 1H, t, *J* = 7.0 Hz), by the HMBC correlations from an olefinic proton H-20 (*δ*_H_ 4.99, 1H, s, *δ*_H_ 4.90, 1H, quint, *J* = 1.4 Hz) to C-18 (*δ*_C_ 66.4) and C-19 (*δ*_C_ 144.3), and by the HMBC correlations from a methyl singlet H-21 (*δ*_H_ 1.82, 3H, s) to C-18 and C-19. The connectivity between the three isoprene units was determined by the HMBC correlations from H-13 to C-12, and C-22 and the COSY crosspeaks between H-16 (*δ*_H_ 2.05, 1H, m, *δ*_H_ 1.95, and 1H, m) and H-17 (Table 1). 

**Figure 2:**
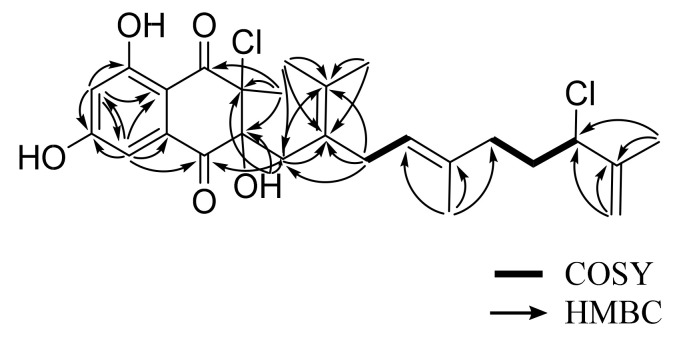
COSY and key HMBC correlations of merochlorin G (**1**).

Lastly, the connection of these two fragments was secured by the interpretation of the HMBC correlations. Correlations from the methylene protons H-11 (*δ*_H_ 2.61, 1H, d, *J* = 14.0 Hz, *δ*_H_ 2.37, 1H, d, *J* =14.0 Hz) to C-1 and from an exchangeable proton 10-OH to C-9, C-10, and C-11 allowed for the attachment of C-10/C-11 and the placement of the hydroxy group at C-10. The HMBC correlations from a methyl singlet H_3_-26 (*δ*_H_ 1.51, 3H, s) to C-8, C-9, and C-10 allowed for the placement of the methyl substituent at C-9. Carbon chemical shifts at *δ*_C_ 74.5 and 66.4; the isotope ratio (9:6:1) of three pseudomolecular ion peaks [M + H]^+^, [M + H + 2]^+^, and [M + H + 4]^+^ in the LR-ESI-MS spectroscopic data; and the fact that there is no other available heteroatom besides two chlorines allowed for the attachment of two chlorine atoms at C-9 and C-18, respectively, thus completing the structural assignment of **1** (Figure 2).

Merochlorin H (**2**) was isolated as a pale-yellow oil, and its molecular formula was assigned as C_26_H_34_^35^Cl_2_O_6_ based on HRESIMS data analysis (a pseudomolecular ion peak at *m/z* 535.1627 [M +Na]^+^, calcd for C_26_H_34_^35^Cl_2_O_6_ Na, 535.1630). The isotope ratio (9:6:1) of three pseudomolecular ion peaks [M + H]^+^, [M + H + 2]^+^, and [M + H + 4]^+^ in the LR-ESI-MS spectrum also supported the presence of two chlorine atoms in the molecule. The ^1^H NMR spectrum of **2** was almost identical to that of **1**, except for the terminal structure of the rearranged sesquiterpene moiety in the terminal fragment. The largest difference in the ^1^H NMR spectrum was attributed to the methyl singlet proton signal H_3_-20 (*δ*_H_ 1.12, 3H, s) instead of the geminal olefinic protons in **1**. The long-ranged HMBC correlations from H_3_-20 and H_3_-21 (*δ*_H_ 1.18, 6H, s) to carbons C-18 (*δ*_C_ 69.8) and C-19 (*δ*_C_ 71.7) and the carbon chemical shift of C-19 indicated that **2** has a hydroxy group at C-19 (Table 1). 

Merochlorin I (**3**) was isolated as a pale-yellow oil, and its molecular formula was assigned as C_26_H_33_^35^Cl_3_O_5_ based on the observation of a pseudomolecular ion peak at *m/z* 553.1281 [M + Na]^+^ in the HRESIMS spectroscopic data (calcd for C_26_H_34_^35^Cl_3_O_5_Na, 553.1291). The ^1^H and ^13^C NMR data for **3** were almost identical to those of **2**, suggesting that they share the same carbon backbone. Comparisons and interpretation of the 2D NMR spectroscopic data of **3** to those of **2** indicated that **3** also possesses dihydronaphthalenedione and rearranged sesquiterpene moieties. The isotope ratio (27:27:9:1) of the four pseudomolecular ion peaks [M + H]^+^, [M + H + 2]^+^, [M + H + 4]^+^, and [M + H + 6]^+^ in the LR-ESI-MS clearly indicated the presence of three chlorines in **3**. The consideration of the carbon chemical shift of C-19 (*δ*_C_ 72.9) and the molecular formula of **3** allowed for the attachment of a chlorine atom at C-19 (Table 1). 

The molecular formula of merochlorin J (**4**) was determined to be C_26_H_36_^35^ClNO_6_ based on a pseudomolecular ion peak (*m/z* 494.2311 [M + H]^+^ (calcd for C_26_H_35_^35^ClNO_6_, 494.2309)) in the HRESIMS spectrum data and interpretation of ^13^C NMR data. The ^1^H NMR spectrum of **4** displayed a pair of *meta*-coupled aromatic protons at *δ*_H_ 6.81 and 6.62; nine methylenes at *δ*_H_ 2.45, 2.31, 2.23, 1.88, 1.65, 1.64, 1.56, 1.42, and 1.17; one methine at *δ*_H_ 3.98; six methyl singlets at *δ*_H_ 1.81, 1.47, 1.16, 1.14, 1.06, and 1.05; and two exchangeable protons at *δ*_H_ 6.16 and 3.98. The ^13^C and HSQC NMR spectroscopic data revealed two carbonyls at *δ*_C_ 195.3 and 193.6; 10 quaternary carbons at *δ*_C_ 164.1, 134.9, 129.2, 126.4, 107.8, 107.7, 84.0, 83.4, 76.1, and 55.5; three methine carbons at *δ*_C_ 107.7, 107.2, and 81.0; five methylene carbons at *δ*_C_ 39.7, 37.9, 36.3, 26.3, and 25.9; and six methyl carbons at *δ*_C_ 24.7, 22.8, 20.0, 20.0, 19.7, and 18.1.

Analysis of 2D NMR spectroscopic data indicated that **4** has a dihydronaphthalenedione and the common rearranged sesquiterpene side chain. The first fragment, the dihydronaphthalenedione moiety, was constructed by the interpretation of the HMBC correlation from H-3 (*δ*_H_ 6.81, 1H, d, *J* = 2.0 Hz) to C-1 (*δ*_C_ 195.3), C-2 (*δ*_C_ 134.9), C-4 (*δ*_C_ 164.1), C-5 (*δ*_C_ 107.7), and C-7 (*δ*_C_ 107.8); from H-5 (*δ*_H_ 6.62, 1H, d, *J* = 2.0 Hz) to C-3 (*δ*_C_ 107.2), C-4, C-6 (*δ*_C_ 165.2), and C-7; from H_3_-26 (*δ*_H_ 1.81, 3H, s) to C-8 (*δ*_C_ 193.6), C-9 (*δ*_C_ 76.1), and C-10 (*δ*_C_ 84.0); and from the exchangeable proton at 10-OH (*δ*_H_ 6.16, 1H, br s) to C-1, C-9, C-10 and C-11 (*δ*_C_ 37.9).

**Figure 3:**
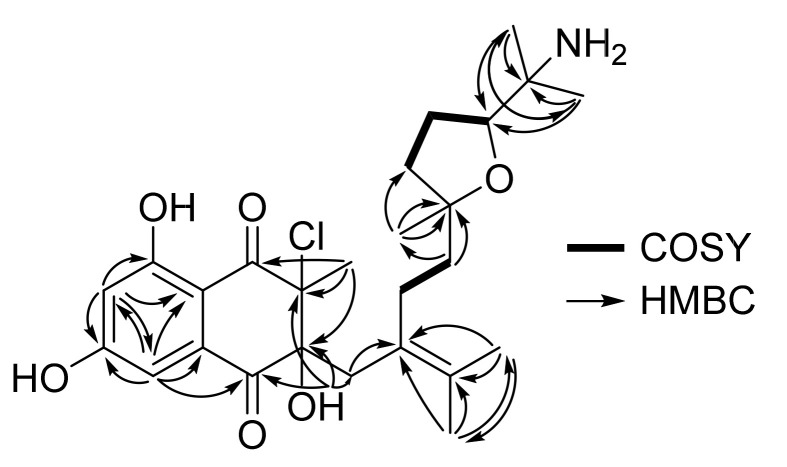
COSY and key HMBC correlations for merochlorin J (**4**).

However, the ^1^H and ^13^C chemical shifts of the sesquiterpene component in **4** were substantially different from those of **1**–**3**. The observation of the COSY crosspeaks for H-16 (*δ*_H_ 1.56, 2H, m)/H-17 (*δ*_H_ 1.88, 1H, m, 1.64, 1H, m)/H-18 (*δ*_H_ 3.98, 1H, t, *J* = 7.6 Hz) and of the HMBC correlations from H_3_-25 (*δ*_H_ 1.06, 3H, s) to C-15 (*δ*_C_ 83.4) and C-16 (*δ*_C_ 36.3); from H_3_-24 (*δ*_H_ 1.47, 3H, s) to C-12 (*δ*_C_ 126.4), C-22 (*δ*_C_ 129.2), and C-23 (*δ*_C_ 20.0); from H_3_-23 (*δ*_H_ 1.05, 3H, s) to C-12, C-23, and C-24 (*δ*_C_ 20.0); from H_3_-21 (*δ*_H_ 1.16, 3H, s) to C-18 (*δ*_C_ 81.0), C-19 (*δ*_C_ 55.5), and C-20 (*δ*_C_ 19.7); from H_3_-20 (*δ*_H_ 1.14, 3H, s) to C-18, C-19, and C-21 (*δ*_C_ 22.8); from H-14 (*δ*_H_ 1.42, 1H, m/*δ*_H_ 1.17, 1H, m) to C-15 and C-25 (*δ*_C_ 24.7); and from H-11 (*δ*_H_ 2.45, 1H, d, *J* = 16.0 Hz/*δ*_H_ 2.31, 1H, d, *J* = 16.0 Hz) to C-12 and the carbon chemical shifts of C-15 and C-18 (*δ*_C_ 81.0) allowed for the construction of the ether linkage between C-15 and C-18 of the second isoprene unit in the sesquiterpene component (Figure 3). Lastly, the consideration of the carbon chemical shift of C-19 (*δ*_C_ 55.5) and the molecular formula of **4** allowed for the attachment of an NH_2_ group at C-19 (Table 2).

Compound **10** was isolated as a dark-yellow oil, and its molecular formula was determined as C_11_H_8_O_5_ based on analysis of HRESIMS data (a pseudomolecular ion peak at *m/z* 220.0373 [M + H]^+^, calcd for C_11_H_8_O_5_, 220.0372). The ^1^H NMR spectrum of **10** was almost identical to that of the first fragment of **1**, except for a methyl singlet at *δ*_H_ 1.89 for H-11. The ^1^H NMR spectrum of **10** displayed two aromatic protons at *δ*_H_ 6.97 (1H, d, *J* = 2.0 Hz, H-3) and 6.54 (1H, d, *J* = 2.0 Hz, H-5), one methyl singlet at *δ*_H_ 1.89 (3H, s, H_3_-11), and two exchangeable protons at *δ*_H_ 12.59 (1H, s, 4-OH) and 10.96 (1H, s, 6-OH). The ^13^C NMR and HSQC spectra of **10** displayed two carbonyl carbons at *δ*_C_ 189.6 (C-8) and 180.0 (C-1); six quaternary carbons at *δ*_C_ 163.4 (C-6), 162.7 (C-4), 155.6 (C-10), 131.8 (C-2), 119.2 (C-9), and 107.2 (C-7); two aromatic carbons at *δ*_C_ 107.9 (C-5) and 107.8 (C-3); and one methyl singlet carbon at *δ*_C_ 7.87 (C-11). Therefore, **10** was identified as 2,5,7-trihydroxy-3-methylnaphthalene-1,4-dione based on the comparison of NMR data in the literature [20]. This compound has been previously reported as a synthesis compound, but it is the first report as a natural product. 

**Figure 4:**
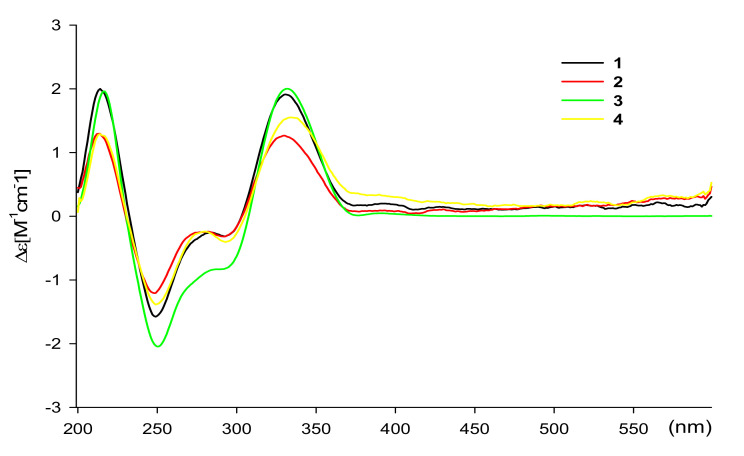
Experimental ECD spectra of **1**–**4** in methanol.

The relative stereochemistry of **1**–**4** was assigned by analysis of NOE spectroscopic data. The NOE crosspeak between 10-OH and the methyl singlet protons H_3_-26 suggested that these protons should be located at the same side on the dihydronaphthalenedione moiety. The chemical structures of **1**−**4** indicated that they are modified biosynthetic products of **7**. This was supported by the conclusion that the C-9 and C-10 stereochemistry of **1**−**4** was likely the same as that of **7**. To determine the absolute configurations of C-9 and C-10 in **1**–**4**, the experimental ECD spectral data of **1**–**4** were measured (Figure 4). The ECD spectra of **1**–**4** exhibited almost identical patterns, suggesting that C-9 and C-10 of the dihydronaphthalenedione moiety in **1**–**4** have the same absolute configurations. Further, comparing their ECD spectra to those of previously reported enantiomer models by our group allowed us to suggest that C-9 and C-10 in **1**–**4** are in 9*S*, 10*R* configurations [19]. Furthermore, compounds **1**, **2**, **3,** and **6** shared the same chemical scaffold and were isolated from the same bacterial strain. These observations suggested that the four compounds likely share the same absolute configuration for C-18. The absolute configurations of C-18 in **1** and of C-15 and C-18 in **4** were determined via DP4 calculations [21]. DP4 calculation is a computational method based on quantum mechanics for confirmation of the absolute configuration using NMR chemical shifts. It was required to design the models of two possible diastereomers of **1** (18*R* and 18*S*) and four possible diastereomer models of **4** (15*R*/18*R*, 15*R*/18*S*, 15*S*/18*R*, and 15*S*/18*S*). As a result, DP4 analysis indicated that **1** had an 18*R* configuration with 86% probability (Figure S22), whereas **4** had 15*R*, 18*R* configurations with a 100% probability (Figure S23).

Previous studies have proposed plausible biosynthetic pathways for tetrahydroxynaphthalene (THN)-derived meroterpenes from strain CNH-189, such as merochlorins, ansalactams, and meroindenon [17,18,19]. These pathways started from THN coupled with a C_15_ isoprene unit with atypical modifications, including a Baeyer‒Villiger-style oxidation, a Paterno‒Büchi-type 2 + 2 cycloaddition, and a pinacol-type contraction. All instances of atypical enzymatic biosynthesis are good examples of the biosynthetic potential of this marine microorganism.

There are two classes of bacterial THN-derived meroterpenoids, depending on the attachment of an isoprene chain to the THN frame [22]. Class I includes neomarinone, merochlorin A (**5**), and merochlorin B, which have a modified isoprene unit at the C-2 or C-4 carbon of the THN [23]. Class II compounds include merochlorins G–J (**1**–**4**) and are characterized by the attachment of an isoprene unit at the C-3 carbon of THN. Compounds **1**, **2**, and **3** are chlorinated products of merochlorin D (**7**), and compound **4** is an intramolecular cyclization product with further amination at C-19. Interestingly, chlorination at the isoprene chain of **1**–**3** occurred specifically at C-18. A tetrahydrofuran ring moiety identified in **4** was likely a biosynthesis product of an epoxide-opening reaction that starts with the selective enzymatic epoxidation of the double bonds [23,24,25]. Based on an AntiMarin2013 database search, the amine group at the tail of the isoprene chain of **4** was the first reported marine microbial natural product to possess this modification. It is also a rare case of an amination product from a tertiary alcohol that was produced by an epoxide-opening reaction. There are some synthetic examples of amination in the isopropyl group [26]. Lastly, compound **10** could be synthesized from the reduction of THN to hydroxynaphthoquinone, followed by methylation at C-3 [18,27].

Compounds **1**–**10** were tested for their antibacterial activities against six pathogenic bacteria, including three Gram-positive bacteria (*Bacillus subtilis* KCTC 1021, *Kocuria rhizophila* KCTC 1915, and *Staphylococcus aureus* KCTC 1927) and three Gram-negative bacteria (*Escherichia coli* KCTC 2441, *Salmonella typhimurium* KCTC 2515, and *Klebsiella pneumoniae* KCTC 2690). Merochlorin I (**3**) displayed strong antibacterial activities against *B. subtilis*, *K. rhizophila*, and *S. aureus*, with MIC values of 1, 2, and 2 μg/mL, respectively. Merochlorin G (**1**) was found to exhibit moderate antibacterial activities against the pathogenic strains, with a 16–32 μg/mL MIC range. Compound **10** exhibited strong antibacterial activity against *K. rhizophila* and moderate activity against *B. subtilis*, with MIC values of 2 and 32 μg/mL, respectively. Meanwhile, merochlorins H (**2**) and J (**4**) did not show any significant antibacterial activities against the six tested pathogens, with MIC values up to 128 μg/mL (Table 3). The presence of a polar moiety at the isoprene chain (C-19) abrogated the antibacterial properties of merochlorins, which was demonstrated with the hydroxy group in **2** and the amine group in **4**. This was also supported by the strong antibacterial activity of **7**–**9,** which possess nonpolar isoprene chain moieties. Therefore, the antibacterial activity against Gram-positive bacteria of these compounds depended on the hydroxy group at the THN core [28,29]. Interestingly, merochlorin A (**5**) exhibited no antibacterial activity against *K. rhizophila*, with MIC values up to 128 μg/mL, but exerted a strong antibacterial activity against two other Gram-positive bacteria (*B. subtilis* and *S. aureus*) [30].

## 3. Materials and Methods

### 3.1. General Experimental Procedures

Optical rotations were acquired using a Kruss Optronic P-8000 polarimeter with a 5 cm cell. UV spectra were recorded in a Varian Cary UV-visible spectrophotometer with a path length of 1 cm, and IR spectra were recorded on a Perkin-Elmer 1600 FT-IR spectrometer. Low-resolution LC/MS measurements were performed using the Agilent Technologies 1260 quadrupole and Waters Micromass ZQ LC/MS system using a reversed-phase column (Phenomenex Luna C18 (2) 100 Å, 50 mm × 4.6 mm, 5 *µ*m) at a flow rate of 1.0 mL/min at the National Research Facilities and Equipment Center (NanoBioEnergy Materials Center) at Ewha Womans University. CD spectra were recorded using an Applied Photophysics Chirascan-Plus circular dichroism spectrometer (Applied Photophysics Ltd., Leatherhead, Surrey, UK). ^1^H and 2D NMR spectra data were recorded at 400 and 800 MHz in DMSO-*d_6_* and CDCl_3_ solutions containing Me_4_Si as an internal standard on Varian Inova spectrometers. ^13^C NMR spectra were acquired at 100 or 200 MHz on a Varian Inova spectrometer. High-resolution EI-MS spectra were acquired using a JEOL JMS-AX505WA mass spectrometer at Seoul National University. 

### 3.2. Collection and Phylogenetic Analysis of Strain CNH-189 

The marine-derived actinomycete strain CNH-189 was isolated from a marine sediment sample collected near Oceanside, California. The strain was identified as a *Streptomyces* sp. based on 16S rRNA gene sequence analysis (accession no. HQ214120).

### 3.3. Fermentation

The bacterium strain CNH-189 was cultured in 60 2.8 L Fernbach flasks, each containing 1 L of a deionized water-based medium (M1: 10 g/L glucose, 20 g/L Grandma’s molasses, 5 g/L peptone, 2 g/L CaCO_3_, 40 mg/L Mg(SO_4_)·4H_2_O, 200 mg/L KCl, 200 mg/L KBr, and 40 mg/L Fe_2_(SO_4_)_3_·4H_2_O) at 27 °C. 

### 3.4. Extraction and Isolation

Sterilized XAD7HP resin (20 g/L) was added after 24 h of cultivation, and the culture was incubated for an additional 5 days at 27 °C. Once the bacteria were cultured in the presence of XAD7HP resin, the resin was collected on cheesecloth, washed with deionized water, and eluted with acetone. The acetone was removed under reduced pressure, and the resulting aqueous layer was extracted with ethyl acetate (3 × 500 mL). The ethyl-acetate-soluble fraction was dried in vacuo to yield 4.5 g of crude extract. The crude extract was fractionated by open-column chromatography on silica gel (25 g) and then eluted with a step gradient of dichloromethane and methanol to obtain seven fractions. The first fraction was purified by reversed-phase HPLC (Phenomenex Luna C-18(2), 250 × 100 mm^2^, 2.0 mL/min, 5 μm, 100 Å, UV = 254 nm) using an isocratic solvent system to 90% CH_3_CN in water to render merochlorins G (**1**, 4.2 mg, *t*_R_ = 26.1 min) and H (**2**, 3.0 mg, *t*_R_ = 23.2 min). The fourth fraction was subjected to reversed-phase HPLC chromatography eluting with 82% CH_3_CN in water to obtain merochlorin I (**3**, 5.8 mg, *t*_R_ = 30.2 min). The third fraction was purified using 64% CH_3_CN in water to obtain merochlorin J (**4**, 2.0 mg, *t*_R_ = 31.2 min). To isolate compound **10**, strain CNH-189 was regrown in 80 L scale. The treatment of the cultivation and crude extract fractionation was the same as the first culture. The first fraction was purified by reversed-phase HPLC (Phenomenex Luna C-18(2), 250 × 100 mm^2^, 2.0 mL/min, 5 μm, 100 Å, UV = 254 nm) using an isocratic solvent system from 82% CH_3_CN in water to obtain compound **10** (3.3 mg, *t*_R_ = 6.5 min). Merochlorins A (**5**, 2.6 mg, *t*_R_ = 29.1 min), C (**6**, 2.1 mg, *t*_R_ = 19.8 min), D (**7**, 2.2 mg, *t*_R_ = 35.6 min), E (**8**, 3.1 mg, *t*_R_ = 20.3 min), and F (**9**, 2.3 mg, *t*_R_ = 21.1 min) were isolated from the crude extract using a previously reported isolation procedure [19].

*Merochlorin G (**1**)*: white powder; ${\left[\alpha \right]}_{D}^{21}\;$+30 (*c* 0.30, MeOH); UV (MeOH) λ_max_ (log *ε*) 224 (4.70), 239 (4.30), 296 (4.30), 334 (4.30) nm; IR (KBr) *ν*_max_ 3625, 3191, 2924, 2361, 1643, 1376, 1266, 1165, 757 cm^−1^; ^1^H and ^13^C NMR (800 MHz and 200 MHz, CDCl_3_), see Table 1; HR-FAB-MS *m*/*z* 517.1517 [M + Na]^+^ (calcd for C_26_H_32_^35^Cl_2_O_5_Na, 517.1524). 

*Merochlorin H (**2**)*: pale-yellow oil; ${\left[\alpha \right]}_{D}^{21}$ +42 (*c* 0.40, MeOH); UV (MeOH) λ_max_ (log *ε*) 224 (4.60), 239 (4.70), 296 (4.30), 334 (4.30) nm; IR (KBr) *ν*_max_ 3562, 3483, 2361, 2066, 1623, 678 cm^−1^; ^1^H and ^13^C NMR (400 MHz and 100 MHz, DMSO-*d_6_*), see Table 1; HR-FAB-MS *m*/*z* 535.1627 [M + Na]^+^ (calcd for C_26_H_34_^35^Cl_2_O_6_ Na, 535.1630). 

*Merochlorin I (**3**)*: pale-yellow oil; ${\left[\alpha \right]}_{D}^{21}$ +85 (*c* 0.60, MeOH); UV (MeOH) λ_max_ (log *ε*) 224 (4.60), 239 (4.70), 296 (4.30), 334 (4.30) nm; IR (KBr) *ν*_max_ 3461, 2932, 2348, 1630, 1369, 1272, 1168, 858, 754 cm^−1^; ^1^H and ^13^C NMR (400 MHz and 100 MHz, DMSO-*d_6_*), see Table 1; HR-FAB-MS *m*/*z* 553.1281 [M + Na]^+^ (calcd for C_26_H_34_^35^Cl_3_O_5_Na, 553.1291). 

*Merochlorin J (**4**)*: pale-yellow oil; ${\left[\alpha \right]}_{D}^{21}$ +68 (*c* 0.275, MeOH); UV (MeOH) λ_max_ (log *ε*) 224 (4.60), 239 (4.70), 296 (4.30), 334 (4.30) nm; IR (KBr) *ν*_max_ 2958, 2357, 1630, 1180, 753 cm^−1^; ^1^H and ^13^C NMR (400 MHz and 100 MHz, DMSO-*d_6_*), see Table 2; HR-FAB-MS *m*/*z* 494.2311 [M + H]^+^ (calcd for C_26_H_35_^35^ClNO_6_, 494.2309). 

*Merochlorin A* (***5***): ^1^H (500 MHz, DMSO-*d_6_*); *δ*_H_ 11.9 (br s, 3-OH), 6.16 (d, *J* = 2.0 Hz, H-4), 6.38 (d, *J* = 2.0 Hz, H-6), 2.24 (dd, *J* = 9.4, 4.0 Hz, H-9), 2.87 (d, *J* = 13.0 Hz, H-13)/2.65 (d, *J* = 13.0 Hz, H-13), 2.36 (dd, *J* = 14.0, 4.0 Hz, H-15)/2.33 (dd, *J* = 14.0, 9.4 Hz, H-15), 1.14 (q, *J* = 6.0 Hz, H-16)/1.40 (dt, *J* = 14.8, 4.8 Hz, H-16), 2.03 (m, H-17)/1.75 (m, H-17), 4.92 (t, *J* = 6.5 Hz, H-18), 1.45 (s, H-20), 1.53 (s, H-21), 1.56 (s, H-22), 1.65 (s, H-24), 0.81 (s, H-25), ^13^C NMR (125 MHz, DMSO-*d_6_*); *δ*_C_ 200.1 (C-12), 193.2 (C-1), 166.5 (C-5), 165.4 (C-3), 150.5 (C-7), 132.1 (C-14), 131.6 (C-19), 124.2 (C-18), 123.1 (C-23), 109.8 (C-2), 102.1 (C-4), 103.7 (C-6), 91.3 (C-11), 61.5 (C-8), 58.8 (C-9), 45.3 (C-10), 39.2 (C-16), 31.9 (C-15), 29.3 (C-13), 26.1 (C-21), 22.8 (C-17), 21.1 (C-24), 20.9 (C-22), 18.1 (C-20), 16.5 (C-25), LR-ESI-MS *m/z* = 429.18 [M + H]^+^. 

*Merochlorin C (**6**)*: ^1^H (500 MHz, DMSO-*d_6_*); *δ*_H_ 11.2 (s, 6-OH), 5.82 (s, 10-OH), 7.19 (d, *J* = 2.0 Hz, H-3), 6.91 (d, *J* = 2.0 Hz, H-5), 4.36 (t, *J* = 4.8 Hz, H-14), 3.84 (d, *J* = 10.2 Hz, H-18), 2.88 (d, *J* = 16.0 Hz, H-11)/2.11 (d, *J* = 16.0 Hz, H-11), 2.79 (d, *J* = 14.0 Hz, H-13)/1.81 (m, H-13), 1.96 (m, H-16), 1.76 (s, H-22), 1.74 (s, H-26), 1.70 (s, H-21), 1.61 (m, H-17)/1.54 (m, H-17), 1.59 (s, H-24), 1.58 (s, H-20), 0.98 (s, H-25), ^13^C NMR (125 MHz, DMSO-*d_6_*); *δ*_C_ 196.4 (C-1), 187.1 (C-8), 164.0 (C-6), 159.0 (C-4), 136.2 (C-2), 135.1 (C-15), 132.5 (C-23), 124.9 (C-14), 124.5 (C-12), 118.9 (C-5), 118.8 (C-7), 110.6 (C-3), 87.8 (C-19), 84.2 (C-10), 78.0 (C-9), 66.4 (C-18), 38.2 (C-11), 35.8 (C-16), 31.8 (C-17), 30.5 (C-13 & C-21), 25.4 (C-20), 22.6 (C-22), 21.1 (C-24), 19.9 (C-26), 16.5 (C-25), LR-ESI-MS *m/z* = 517.15 [M + Na]^+^.

*Merochlorin D (**7**)*: ^1^H (500 MHz, DMSO-*d_6_*); *δ*_H_ 11.5 (s, 6-OH), 6.09 (s, 10-OH), 6.83 (d, *J* = 2.0 Hz, H-3), 6.60 (d, *J* = 2.0 Hz, H-5), 4.97 (d, *J* = 6.0 Hz, H-18), 4.77 (t, *J* = 6.0 Hz, H-14), 2.82 (dd, *J* = 14.0, 6.0 Hz, H-13)/2.35 (dd, *J* = 14.0, 6.0 Hz, H-13), 2.42 (d, *J* = 16.0 Hz, H-11)/2.24 (d, *J* = 16.0 Hz, H-11), 1.95 (m, H-17), 1.84 (m, H-16), 1.77 (s, H-26), 1.59 (s, H-20), 1.51 (s, H-21), 1.47 (s, H-22), 1.42 (s, H-25), 1.08 (s, H-24), ^13^C NMR (125 MHz, DMSO-*d_6_*); *δ*_C_ 195.9 (C-1), 194.1 (C-8), 166.0 (C-4), 165.0 (C-6), 135.7 (C-2), 134.9 (C-15), 131.3 (C-19), 130.3 (C-23), 126.5 (C-12), 124.6 (C-18), 123.1 (C-14), 108.6 (C-7), 108.4 (C-5), 108.1 (C-3), 84.5 (C-10), 77.0 (C-9), 39.0 (C-11), 39.6 (C-16), 31.1 (C-13), 26.8 (C-20), 26.7 (C-17), 21.3 (C-24), 21.0 (C-22), 18.9 (C-26), 18.8 (C-21), 16.8 (C-25), LR-ESI-MS *m/z* = 483.19 [M + Na]^+^.

*Merochlorin E (**8**)*: ^1^H (500 MHz, DMSO-*d_6_*); *δ*_H_ 11.52 (s, 4-OH), 11.47 (s, 6-OH), 5.95 (s, 10-OH), 6.85 (d, *J* = 2.0 Hz, H-3), 6.63 (d, *J* = 2.0 Hz, H-5), 5.02 (t, *J* = 4.8 Hz, H-16), 2.69 (d, *J* = 14.1 Hz, H-11)/2.12 (d, *J* = 14.1 Hz, H-11), 2.23 (dd, *J* = 14.0, 4.8 Hz, H-13)/1.22 (m, H-13), 1.80 (s, H-26), 1.78 (m, H-17)/1.63 (m, H-17), 1.52 (s, H-24), 1.36 (s, H-25), 1.33 (s, H-22), 1.05 (dd, *J* = 14.0, 14.0 Hz, H-18)/0.88 (dd, *J* = 14.0, 14.0 Hz, H-18), 0.82 (s, H-20), 0.72 (s, H-21), ^13^C NMR (125 MHz, DMSO-*d_6_*); *δ*_C_ 195.2 (C-1), 193.4 (C-8), 165.6 (C-6), 164.1 (C-4), 136.6 (C-15), 135.2 (C-2), 130.9 (C-23), 125.1 (C-12), 119.4 (C-16), 107.8 (C-7), 107.6 (C-5), 107.3 (C-3), 83.9 (C-10), 78.0 (C-9), 47.0 (C-14), 39.5 (C-11), 34.4 (C-13), 32.0 (C-19), 28.6 (C-18), 28.1 (C-20), 26.3 (C-21), 24.8 (C-25), 22.5 (C-17), 20.8 (C-22 & C-24), 18.1 (C-26), LR-ESI-MS *m/z* = 461.20 [M + H]^+^.

*Merochlorin F (**9**)*: ^1^H (500 MHz, DMSO-*d_6_*); *δ*_H_ 11.52 (s, 4-OH), 11.47 (s, 10-OH), 5.99 (s, 10-OH), 6.83 (d, *J* = 2.0 Hz, H-3), 6.62 (d, *J* = 2.0 Hz, H-5), 5.19 (t, *J* = 4.8 Hz, H-16), 2.56 (d, *J* = 14.0 Hz, H-11)/2.32 (d, *J* = 14.0 Hz, H-11), 2.14 (dd, *J* = 14.8, 14.8 Hz, H-13)/1.78 (m, H-13), 1.91 (m, H-17), 1.48 (m, H-18)/1.01 (dd, *J* = 7.2, 7.2 Hz, H-18), 1.79 (s, H-26), 1.49 (s, H-24), 1.43 (s, H-25), 1.17 (s, H-22), 0.71 (s, H-20), 0.62 (s, H-21), ^13^C NMR (125 MHz, DMSO-*d_6_*); *δ*_C_ 195.1 (C-1), 193.2 (C-8), 165.7 (C-6), 164.1 (C-4), 136.8(C-15), 134.9 (C-2), 130.5 (C-23), 125.7 (C-12), 119.9 (C-16), 107.9 (C-7), 107.7 (C-5), 107.4 (C-3), 83.9 (C-10), 76.0 (C-9), 46.9 (C-14), 38.4 (C-11), 33.7 (C-13), 32.1 (C-19), 29.1 (C-18), 27.9 (C-20), 26.4 (C-21), 24.7 (C-25), 22.6 (C-17), 20.7 (C-22 & C-24), 18.0 (C-26), LR-ESI-MS *m/z* = 461.20 [M + H]^+^.

*Compound **10***: ^1^H (400 MHz, DMSO-*d_6_*); *δ*_H_ 12.59 (s, 4-OH), 10.96 (s, 6-OH), 6.97 (d, *J* = 2.0 Hz, H-3), 6.54 (d, *J* = 2.0 Hz, H-5), 1.89 (s, H-11), ^13^C NMR (100 MHz, DMSO-*d_6_*); *δ*_C_ 189.6 (C-8), 180.0 (C-1), 163.4 (C-6), 162.7 (C-4), 155.6 (C-10), 131.8 (C-2), 119.2 (C-9), 107.9 (C-5), 107.8 (C-3), 107.2 (C-7), 7.8 (C-11), HR-FAB-MS *m*/*z* 220.0373 [M + H]^+^ (calcd for C_11_H_8_O_5_, 220.0372).

### 3.5. Conformational Search and DP4 Calculations 

A conformational search of merochlorins G (**1**) and J (**4**) was carried out by MacroModel with the Merck molecular force field (gas phase), a 10 kJ/mol upper energy limit, and a 0.001 kJ (mol Å)^−1^ convergence threshold on the rms gradient to minimize computational complexity and expense. In the case of **1**, 66 conformers were obtained for the 18*R* diastereomer and 37 conformers were obtained for the 18*S* model under the 10 kJ/mol limit of molecular potential energy. In addition, in the case of **4**, 10 conformers were obtained for the 15*R*/18*R* diastereomer, 5 conformers for the 15*R*/18*S* diastereomer, 2 conformers for the 15*S*/18*R* diastereomer, and 7 conformers for the 15*S*/18*S* diastereomer. The Boltzmann population was calculated based on the potential energy of each conformer. Ground-state geometry optimization was performed by density functional theory (DFT) modeling with TurbomoleX 4.3.2. The basis set was def-SV(P) for all atoms, and the level of theory was B3-LYP at the functional level in the gas phase. Calculated chemical shifts of ^1^H and ^13^C were averaged by the Boltzmann populations. The experimental chemical shifts compared to these Boltzmann-averaged chemical shifts and the DP4 analyses indicated the 18*R* configuration of **1** with 86.6% and 15*R*/18*R* configurations of **4** with 100.0% probability.

### 3.6. Antibacterial Assay

Antibacterial susceptibility was tested against three Gram-positive bacteria (*Bacillus subtilis* KCTC 1021, *Kocuria rhizophila* KCTC 1915, and *Staphylococcus aureus* KCTC 1927) and three Gram-negative bacteria (*Escherichia coli* KCTC 2441, *Salmonella typhimurium* KCTC 2515, and *Klebsiella pneumoniae* KCTC 2690), as described in a previous study with modifications [16]. These bacteria were grown in Mueller–Hinton broth at 37 °C and 225 rpm for 24 h. Compounds **1**–**10** and positive controls were dissolved in DMSO, and 100 μL of each solution was dispensed into the wells of 96-well plates starting at 128 μg/mL concentration. Compounds **1**–**10** and positive controls were serially diluted, and Mueller–Hinton broth was added to a final concentration of 0.5% McFarland standard. The 96-well microtiter plates were cultivated for 24 h at 37 °C. The minimal inhibitory concentration (MIC) was defined as the lowest positive control concentration that visibly inhibited bacterial growth [31]. 

## 4. Conclusions

In conclusion, an intensive chemical screening of the *Streptomyces* sp. strain CNH-189 resulted in the discovery of new merochlorin derivatives (**1**–**4**) and a proposed biosynthetic merochlorin precursor **10**, along with known congeners **5**–**9**. Further, compound **3** exhibited strong antibacterial activities against Gram-positive strains such as *B. subtilis*, *K. rhizophila*, and *S. aureus*, with MIC values of 1–2 μg/mL.

## Figures and Tables

**Table 1:** NMR spectroscopic data for merochlorin G (**1**) in CDCl_3_ and ^1^H and ^13^C NMR spectroscopic data for merochlorins H and I (**2** and **3**) in DMSO-*d*_6._

No.	1 ^a^				2 ^b^			3 ^b^	
	*δ*_C_, Mult. ^c^	*δ*_H_ (*J* in Hz)	COSY	HMBC	*δ*_C_, Mult. ^c^	*δ*_H_ (*J* in Hz)	*δ*_C_, Mult. ^c^		*δ*_H_ (*J* in Hz)
1	196.1, C				195.2, C		195.1, C		
2	134.7, C				135.1, C		135.0, C		
3	107.4, CH	7.01, d (2.0)	H-5	1, 2, 4, 5, 7	107.3, CH	6.84, d (2.0)	107.3, CH		6.94, d (2.0)
4	162.9, C				165.7, C		164.2, C		
5	108.9, CH	6.71, d (2.0)	H-3	3, 4, 6, 7	107.8, CH	6.64, d (2.0)	107.7, CH		6.62, d (2.0)
6	165.3, C				164.2, C		165.7, C		
7	109.9, C				108.0, C		108.0, C		
8	194.8, C				193.4, C		193.3, C		
9	74.5, C				76.2, C		76.2, C		
10	84.0, C				84.0, C		84.0, C		
11	38.6, CH_2_	2.61, d (14.0), 2.37, d (14.0)		1, 13	30.9, CH_2_	1.38, s	31.1, CH_2_		2.13, m
12	131.6, C				125.9, C		125.7, C		
13	31.3, CH_2_	2.84, dd (15.2, 6.7), 2.45, dd (15.2, 6.7)	H-14	11, 14, 24	30.3, CH_2_	2.88, dd (15.2, 7.2), 2.42, dd (15.2, 7.2)	30.3, CH_2_		2.86, dd (15.2, 7.2), 2.40, dd (15.2, 7.2)
14	123.5, CH	4.87, t (7.0)	H-13	13, 16, 24, 26	123.2, CH	4.89, t (7.0)	123.9, CH		4.90, t (7.0)
15	134.0, C				133.5, C		132.9, C		
16	36.6, CH_2_	2.05, m ^d^, 1.95, m ^d^	H-17	14, 17, 18, 26	36.8, CH_2_	2.15, m ^d^, 1.95, m ^d^	36.3, CH_2_		2.18, m ^d^, 2.00, m ^d^
17	34.8, CH_2_	1.92, m ^d^, 1.87, m ^d^	H-16, H-18	16, 18	31.3, CH_2_	1.28, d (9.2)	39.2, CH_2_		2.45, d (14.6), 2.27, d (14.6)
18	66.4, CH	4.33, t (7.0)	H-17	17, 20, 21	69.8, CH	3.51, t (2.0)	70.7, CH		3.93, t (2.0)
19	144.3, C				71.7, C		72.9, C		
20	114.2, CH_2_	4.99, s, 4.90, quint (1.4)		18, 21	24.4, CH_3_	1.12, s	29.1, CH_3_		1.59, s
21	17.0, CH_3_	1.82, s		18, 19	27.6, CH_3_	1.18, s	30.3, CH_3_		1.63, s
22	125.1, C				129.5, C		129.7, C		
23	20.9, CH_3_	1.20, s		10, 11, 12, 13, 14, 22, 24	20.3, CH_3_	1.05, s	20.7, CH_3_		1.09, s
24	20.6, CH_3_	1.58, s		10, 11, 12, 22, 23	20.2, CH_3_	1.49, s	20.3, CH_3_		1.49, s
25	18.1, CH_3_	1.94, s		17	15.6, CH_3_	1.47, s	15.4, CH_3_		1.45, s
26	15.9, CH_3_	1.51, s		8, 9, 10	18.0, CH_3_	1.80, s	18.1, CH_3_		1.79, s
4-OH		5.77, s		3, 4, 5		11.43, s			11.48, s
6-OH		11.70, s		4, 5, 6, 7, 8		11.52, s			11.52, s
10-OH		4.08, s		9, 10, 11		6.14, s			6.13, s

^a^ 800 MHz for ^1^H NMR and 200 MHz for ^13^C NMR. ^b^ 400 MHz for ^1^H NMR and 100 MHz for ^13^C NMR. ^c^ Numbers of attached protons were determined by analysis of 2D spectra. ^d^ Signals were overlapping.

**Table 2:** NMR spectroscopic data for merochlorin J (**4**) in DMSO-*d*_6_ ^a^.

No.	4			
	*δ*_C_, Mult. ^b^	*δ*_H_ (*J* in Hz)	COSY	HMBC
1	195.3, C			
2	134.9, C			
3	107.2, CH	6.81, d (2.0)	H-5	1, 2, 4, 5, 7
4	164.1, C			
5	107.7, CH	6.62, d (2.0)	H-3	3, 4, 6, 7
6	165.2, C			
7	107.8, C			
8	193.6, C			
9	76.1, C			
10	84.0, C			
11	37.9, CH_2_	2.45, d (16.0), 2.31, d (16.0)		9, 10, 12, 13, 23
12	126.4, C			
13	26.3, CH_2_	2.23, d (14.0), 1.65, m ^c^	H-14	11, 23
14	39.7, CH_2_	1.42, m ^c^, 1.17, m ^c^	H-13	15, 25
15	83.4, C			
16	36.3, CH_2_	1.56, m ^c^	H-17	14, 15, 17, 18, 25
17	25.9, CH_2_	1.88, m ^c^ 1.64, m ^c^	H-16, H-18	15, 16, 18, 19
18	81.0, CH	3.82, t (7.6)	H-17	20, 21
19	55.5, C			
20	19.7, CH_3_	1.14, s		18, 19, 21
21	22.8, CH_3_	1.16, s		18, 19, 20
22	129.2, C			
23	20.0, CH_3_	1.05, s		12, 22, 24
24	20.0, CH_3_	1.47, s		12, 22, 23
25	24.7, CH_3_	1.06, s		14, 15, 16
26	18.1, CH_3_	1.81, s		8, 9, 10
10-OH		6.16, br s		1, 9, 10, 11

^a^ 400 MHz for ^1^H NMR and 100 MHz for ^13^C NMR. ^b^ Numbers of attached protons were determined by analysis of 2D spectra. ^c^ Signals were overlapping.

**Table 3:** Antibacterial activities of merochlorins (**1**–**10**) ^a^.

	MIC (μg/mL)						
	Gram-Positive Bacteria			Gram-Negative Bacteria			
	*B. subtilis* KCTC 1021	*K. rhizophila* KCTC 1915	*S. aureus* KCTC 1927		*E. coli* KCTC 2441	*S. typhimurium* KCTC 2515	*K. pneumonia* KCTC 2690
1	16	32	16		>128	>128	>128
2	64	>128	>128		>128	>128	>128
3	1	2	2		>128	>128	>128
4	>128	>128	>128		>128	>128	>128
5	2	>128	4		>128	>128	>128
6 ^b^	16	32	32		>128	>128	>128
7	1	0.5	1		>128	>128	>128
8 ^b^	1	2	2		>128	>128	>128
9 ^b^	1	2	1		>128	>128	>128
10	32	2	>128	>128	>128	>128	>128
Ampicillin	0.25	0.25	0.25		4	2	>128
Vancomycin	0.25	0.5	0.5		>128	>128	>128

**^a^** Each sample was tested in triplicate and repeated three times. ^b^ Tested in the previous study.

## Data Availability

The data presented in this study are available on request from the corresponding author.

## Supplementary Materials

The following are available online at www.mdpi.com/article/10.3390/md19110618/s1, Figures S1–S21: 1D NMR and 2D NMR of compounds **1**–**4**, **10**; Figures S22 and S23; DP4 analysis of NMR calculation of compounds **1** and **4**; Figures S24–S32; LRESIMS and HRESIMS spectra of compounds **1**−**4**, **10**; Figures S33 and S34; Simulated conformer models of possible diastereomers of compounds **1** and **4**; Tables S1 and S2: Experimental and calculation chemical shift values of compounds **1** and **4**.

## References

[1] Nett M., Ikeda H., Moore B.S. (2009). Genomic Basis for Natural Product Biosynthetic Diversity in the Actinomycetes. Nat. Prod. Rep..

[2] Almasi F., Mohammadipanah F., Adhami H.R., Hamedi J. (2018). Introduction of Marine-Derived *Streptomyces* sp. UTMC 1334 as A Source of Pyrrole Derivatives with Anti-Acetylcholinesterase Activity. J. Appl. Microbiol..

[3] Mohammadipanah F., Matasyoh J., Hamedi J., Klenk H.P., Laatsch H. (2012). Persipeptides A and B, Two Cyclicpeptides from *Streptomyces* sp. UTMC 1154. Bioorganic Med. Chem..

[4] Imada C. (2005). Enzyme Inhibitors and Other Bioactive Compounds from Marine Actinomycetes. Antonie Van Leeuwenhoek.

[5] Salimi F., Hamedi J., Motevaseli E., Mohammadipanah F. (2017). Isolation and Screening of Rare-Actinobacteria, A New Insight for Finding Natural Products with Anti-Vascular Calcification Activity. J. Appl. Microbiol..

[6] Salcedo R.l.G.a., Olano C., Gomez C., Fernandez R., Brana A.F., Mendez C., Calle F., Salas J.A. (2017). Characterization and Engineering of the Biosynthesis Gene Cluster for Antitumor Macrolides PM100117 and PM100118 from A Marine Actinobacteria: Generation of A Novel Improved Derivative. Microb. Cell Fact..

[7] Azarakhsh Y., Mohammadipanah F., Nassiri S.M., Siavashi V., Hamedi J. (2017). Isolation and Screening of Proangiogenic and Antiangiogenic Metabolites Producing Rare Actinobacteria from Soil. J. Appl. Microbiol..

[8] Dharmaraj S. (2010). Marine Streptomyces as a Novel Source of Bioactive Substances. World J. Microbiol. Biotechnol..

[9] Prieto-Davó A., Fenical W., Jensen P.R. (2008). Comparative Actinomycete Diversity in Marine Sediments. Aquat. Microb. Ecol..

[10] Udwary D.W., Zeigler L., Asolkar R.N., Singan V., Lapidus A., Fenical W., Jensen P.R., Moore B.S. (2007). Genome Sequencing Reveals Complex Secondary Metabolome in the Marine Actinomycete *Salinispora Tropica*. Proc. Natl. Acad. Sci. USA.

[11] Nam S.J., Kauffman C.A., Paul L.A., Jensen P.R., Fenical W. (2013). Actinoranone, A Cytotoxic Meroterpenoid of Unprecedented Structure from A Marine Adapted *Sterptomyces* sp.. Org. Lett..

[12] Sproule A., Correa H., Decken A., Haltli B., Berrue F., Overy D.P., Kerr R.G. (2019). Terrosamycins A and B, Bioactive Polyether Ionophores from *Streptomyces* sp. RKND004 from Prince Edward Island Sediment. Mar. Drug..

[13] Kim M.C., Cullum R., Hebishy A.M.S., Mohamed H.A., Faraag A.H.I., Salah N.M., Abdelfattah M.S., Fenical W. (2020). Mersaquinone, A New Tetracene Derivative from The Marine-Derived *Streptomyces* sp. EG1 Exhibiting Activity Against Methicillin-Resistance *Staphylococcus aureus* (MRSA). Antibiotics.

[14] Paderog M.J.V., Suarez A.F.L., Sabido E.M., Low Z.J., Saludes J.P., Dalisay D.S. (2020). Anthracycline Shunt Metabolites from Philippine Marine Sediment-Derived Streptomyces Destroy Cell Membrane Integrity of Multidrug-Resistant *Staphylococcus aureus*. Front. Microbiol..

[15] Song Y., Yang J., Yu J., Li J., Yuan J., Wong N., Ju J. (2020). Chlorinated Bis-indole Alkaloids from Deep-Sea Derived *Streptomyces* sp. SCSIO 11791 with Antibacterial and Cytotoxic Activities. J. Antibiot..

[16] Wilson M.C., Nam S.J., Gulder T.A.M., Kauffman C.A., Jensen P.R., Fenical W., Moore B.S. (2011). Structure and Biosynthesis of the Marine *Streptomycete* Ansamycin Ansalactam A and Its Distinctive Branched Chain Polyketide Extender Unit. J. Am. Chem. Soc..

[17] Le T.C., Yang I., Yoon Y.J., Nam S.J., Fenical W. (2016). Ansalactams B-D Illustrate Further Biosynthetic Plasticity within the Ansamycin Pathway. Org. Lett..

[18] Kaysser L., Bernhardt P., Nam S.J., Loesgen S., Ruby J.G., Skewes-Cox P., Jensen P.R., Fenical W., Moore B.S. (2012). Merochlorins A-D, Cyclic Meroterpenoid Antibiotics Biosynthesized in Divergent Pathways with Vanadium-Dependent Chloroperoxidases. J. Am. Chem. Soc..

[19] Ryu M.J., Hwang S., Kim S., Yang I., Oh D.C., Nam S.J., Fenical W. (2019). Meroindenon and Merochlorins e and F, Antibacterial Meroterpenoids from a Marine-Derived Sediment Bacterium of the Genus *Streptomyces*. Org. Lett..

[20] Sabutskii Y.E., Polonik S.G., Denisenko V.A., Dmitrenok P.S. (2014). A New Method for Thiomethylation of Hydroxy-1,4-naphthoquinones with *N*-Acetyl-_L_-cystein; First Synthesis of Fibrostatin, B,C, and D. Synthesis.

[21] Zanardi M.M., Suarez A.G., Sarotti A.M. (2020). Determination of the Relative Configuration of Terminal and Spiroepoxides by Computational Methods. Advantages of the Inclusion of Unscaled Data. J. Org. Chem..

[22] Maxwell A. (1989). and Rampersad, D. Novel Prenylated Hydroxybenzoic Acid Derivatives from *Piper Saltuum*. J. Nat. Prod..

[23] Miles Z.D., Diethelm S., Pepper H.P., Huang D.M., George J.H., Moore B.S. (2017). A Unifying Paradigm for Naphthoquinone-Based Meroterpenoid (Bio)Synthesis. Nat. Chem..

[24] Morimoto Y., Iwai T., Kinoshita T. (2000). Revised Structure of Squalene-Derived PentaTHF Polyether, Glabrescol, through Its Enantioselective Total Synthesis: Biogenetically Intriguing C(s) vs C2 Symmetric Relationships. J. Am. Chem. Soc..

[25] Lorente A., Lamariano-Merketegi J., Albericio F., Álvarez M. (2013). Tetrahydrofuran-Containing Macrolides: A Fascinating Gift from the Deep Sea. Chem. Rev..

[26] Bahn S., Imm S., Neubert L., Zhang M., Neumann H., Beller M. (2011). The Catalytic amination of Alcohols. ChemCatChem..

[27] Saha N., Muller M., Husain S.S. (2019). Asymmetric Synthesis of Natural cis-Dihydroarenediols Using Tetrahydroxynaphthalene Reductase and Its Biosynthetic Implications. Org. Lett..

[28] López-Pérez B., Pepper H.P., Ma R., Fawcett B.J., Pehere A.D., Wei Q., Ji Z., Polyak S.W., Dai H., Song F. (2017). Biosynthetically Guided Structure–Activity Relationship Studies of Merochlorin A, an Antibiotic Marine Natural Product. ChemMedChem.

[29] Yang H., Liu X., Li Q., Li L., Zhang J.R., Tang Y. (2015). Total Synthesis and Preliminary SAR Study of (±)-Merochlorins A and B. Org. Biomol. Chem..

[30] Sakoulas G., Nam S.J., Loesgen S., Fenical W., Jensen P.R., Nizet V., Hensler M. (2012). Novel Bacterial Metabolite Merochlorin A Demonstrates in Vitro Activity against Multi-Drug Resistant Methicillin-Resistant *Staphylococcus aureus*. PLoS ONE.

[31] Wiegand I., Hilpert K., Hancock R.E.W. (2008). Agar and Broth Dilution Methods to Determine the Minimal Inhibitory Concentration (MIC) of Antimicrobial Substances. Nat. Protoc..

