# Supplementary material for: Antibacterial Meroterpenoids, Merochlorins G–J from the Marine Bacterium Streptomyces sp
Source: Mar Drugs. 2021 Oct 30;19(11):618. doi: 10.3390/md19110618 (PMC8624273; doi:10.3390/md19110618)
Supplement: Supplementary file 1 [file marinedrugs-19-00618-s001.zip › marinedrugs-1438765-supplementary.pdf]

Supporting Information for

Antibacterial Meroterpenoids, Merochlorins G–J from the Marine Bacterium *Streptomyces* sp.

Min-Ji Ryu[1], *Prima F. Hillman*[,1], *Jihye Lee*[1,2], *Sunghoon Hwang*[3], *Eun-Young Lee*[1], *Sun-Shin Cha*[1], *Inho Yang*[4], *Dong-Chan Oh*[3], *Sang-Jip Nam*[1,*], *and William Fenical*[5,*]

[1]Department of Chemistry and Nanoscience, Ewha Womans University, Seoul 03760, Korea

[2]Laboratories of Marine New Drugs, REDONE Seoul, Seoul 08594, Korea

[3]Natural Products Research Institute, College of Pharmacy, Seoul National University, Seoul 08826, Korea

[4]Department of Convergence Study on the Ocean Science and Technology, Korea Maritime and Ocean University, Busan 49112, Korea

[5]Center for Marine Biotechnology and Biomedicine, Scripps Institution of Oceanography, University of California San Diego, La Jolla, CA 92093-0204, USA

Table of Contents

Figure S1. ^{1}H NMR spectrum of Merochlorin G (**1**) in $CDCl_3$ …………………………………………………………………..……………. **S5**

Figure S2. ^{13}C NMR Spectrum of Merochlorin G (**1**) in $CDCl_3$ ……………………………………………………………………..…... **S6**

Figure S3. COSY Spectrum of Merochlorin G (**1**) in $CDCl_3$ ……………………………………………………………………….……. **S7**

Figure S4. HSQC Spectrum of Merochlorin G (**1**) in $CDCl_3$ ……………………………………………………………………….……. **S8**

Figure S5. HMBC Spectrum of Merochlorin G (**1**) in $CDCl_3$ ……………………………………………………………………….…. **S9**

Figure S6. NOESY Spectrum of Merochlorin G (**1**) in $CDCl_3$ ………………………………………………………………………… **S10**

Figure S7. ^{1}H NMR Spectrum of Merochlorin H (**2**) in DMSO-d_6 ……………………………………………………….………… **S11**

Figure S8. ^{13}C NMR Spectrum of Merochlorin H (**2**) in DMSO-d_6 ……………………………………………………….……... **S12**

Figure S9. ^{1}H NMR Spectrum of Merochlorin I (**3**) in DMSO-d_6 ……………………………………………………………….. **S13**

Figure S10. ^{13}C NMR Spectrum of Merochlorin I (**3**) in DMSO-d_6 ……………………………………………………………... **S14**

Figure S11. ^{1}H NMR Spectrum of Merochlorin J (**4**) in DMSO-d_6 …………………………………………………….……... **S15**

Figure S12. ^{13}C NMR Spectrum of Merochlorin J (**4**) in DMSO-d_6 ……………………………………………………………... **S16**

Figure S13. COSY Spectrum of Merochlorin J (**4**) in DMSO-d_6 ……………………………………………………………………… **S17**

Figure S14. HSQC Spectrum of Merochlorin J (**4**) in DMSO-d_6 ……………………………………………………………………… **S18**

Figure S15. HMBC Spectrum of Meroindenon J (**4**) in DMSO-d_6 …………………………………………………………….…. **S19**

Figure S16. NOESY Spectrum of Meroindenon J (**4**) in DMSO-d_6 ……………………………………………………………… **S20**

Figure S17. ^{1}H NMR Spectrum of Compound **10** in DMSO-d_6.. **S21**

Figure S18. ^{13}C NMR Spectrum of Compound **10** in DMSO-d_6 .... **S22**

Figure S19. COSY Spectrum of Compound **10** in DMSO-d_6.. **S23**

Figure S20. HSQC Spectrum of Compound **10** in DMSO-d_6.. **S24**

Figure S21. HMBC Spectrum of Compound **10** in DMSO-d_6 .... **S25**

Figure S22. DP4 analysis of NMR calculation of Merochlorin G (**1**) **S26**

Figure S23. DP4 analysis of NMR calculation of Merochlorin J (**4**) **S27**

Figure S24. LRMS spectra of Merochlorins G (**1**) **S28**

Figure S25. LRMS spectrum of Merochlorin H (**2**) **S29**

Figure S26. LRMS spectrum of Merochlorin I (**3**) **S30**

Figure S27. LRMS spectrum of Merochlorin J (**4**) **S31**

Figure S28. HRMS spectrum of Merochlorin G (**1**) **S32**

Figure S29. HRMS spectrum of Merochlorin H (**2**) **S33**

Figure S30. HRMS spectrum of Merochlorin I (**3**) **S34**

Figure S31. HRMS spectrum of Merochlorin J (**4**) **S35**

Figure S32. HRMS spectrum of Compound **10** **S36**

Figure S33. Simulated conformer models of two possible diastereomers of **1** (18*R*, 18*S*) **S37**

Figure S34. Simulated conformer models of four possible diastereomers of **4** (15*R*/18*R*, 15*R*/18*S*, 15*S*/18*R*, and 15*S*/18*S*) …………………… **S37-38**

Table S1. Experimental (Exp.) and calculated (Cal.) chemical shift values (CS, δ) of merochlorin G (**1**) ………………………………………. **S39-40**

Table S2. Experimental (Exp.) and calculated (Cal.) chemical shift values (CS, δ) of merochlorin J (**4**) ……………………………………….. **S40-42**

Figure S1. ^{1}H NMR Spectrum of Merochlorin G (**1**) in $CDCl_3$

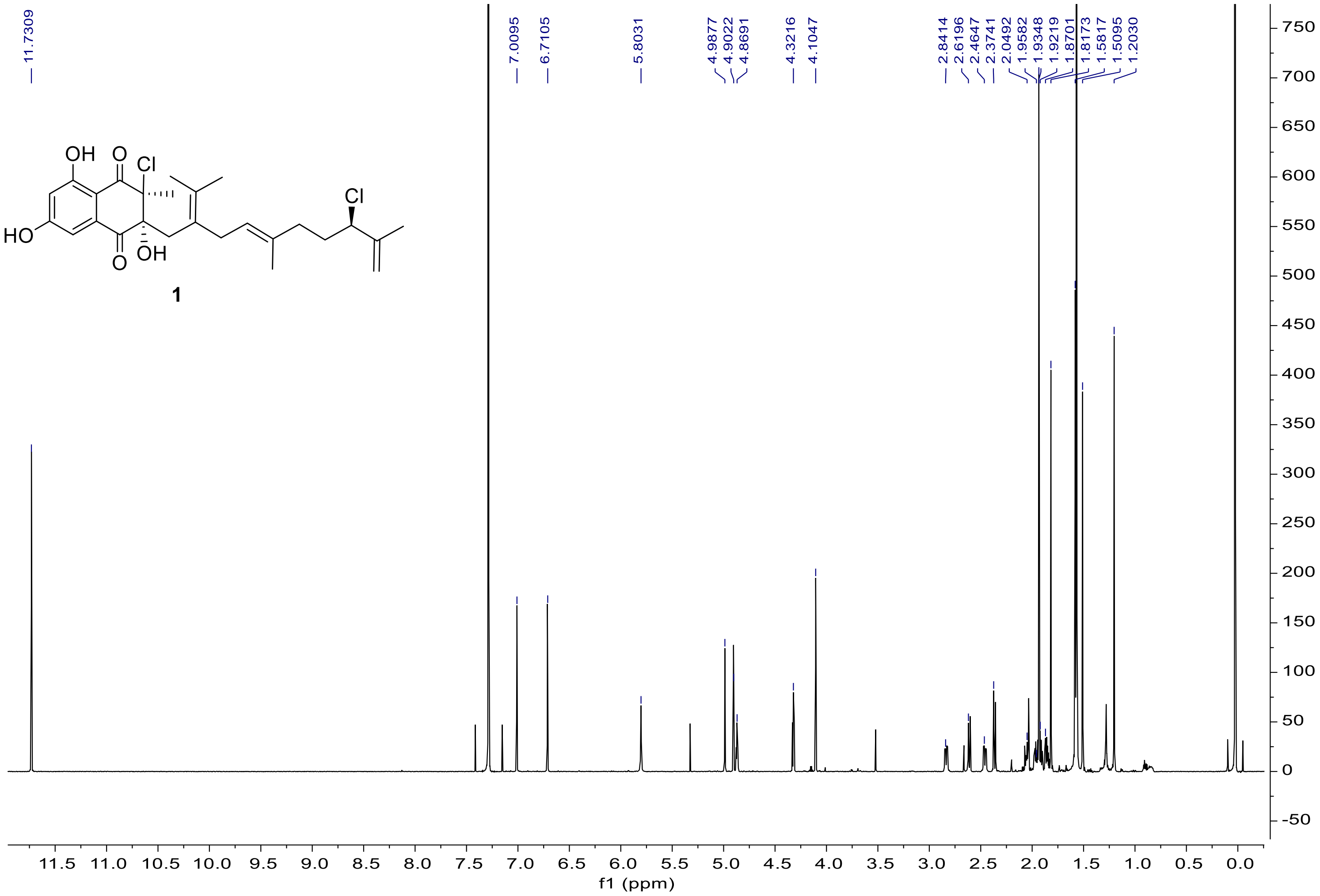

Figure S2. ^{13}C NMR Spectrum of Merochlorin G (**1**) in $CDCl_3$

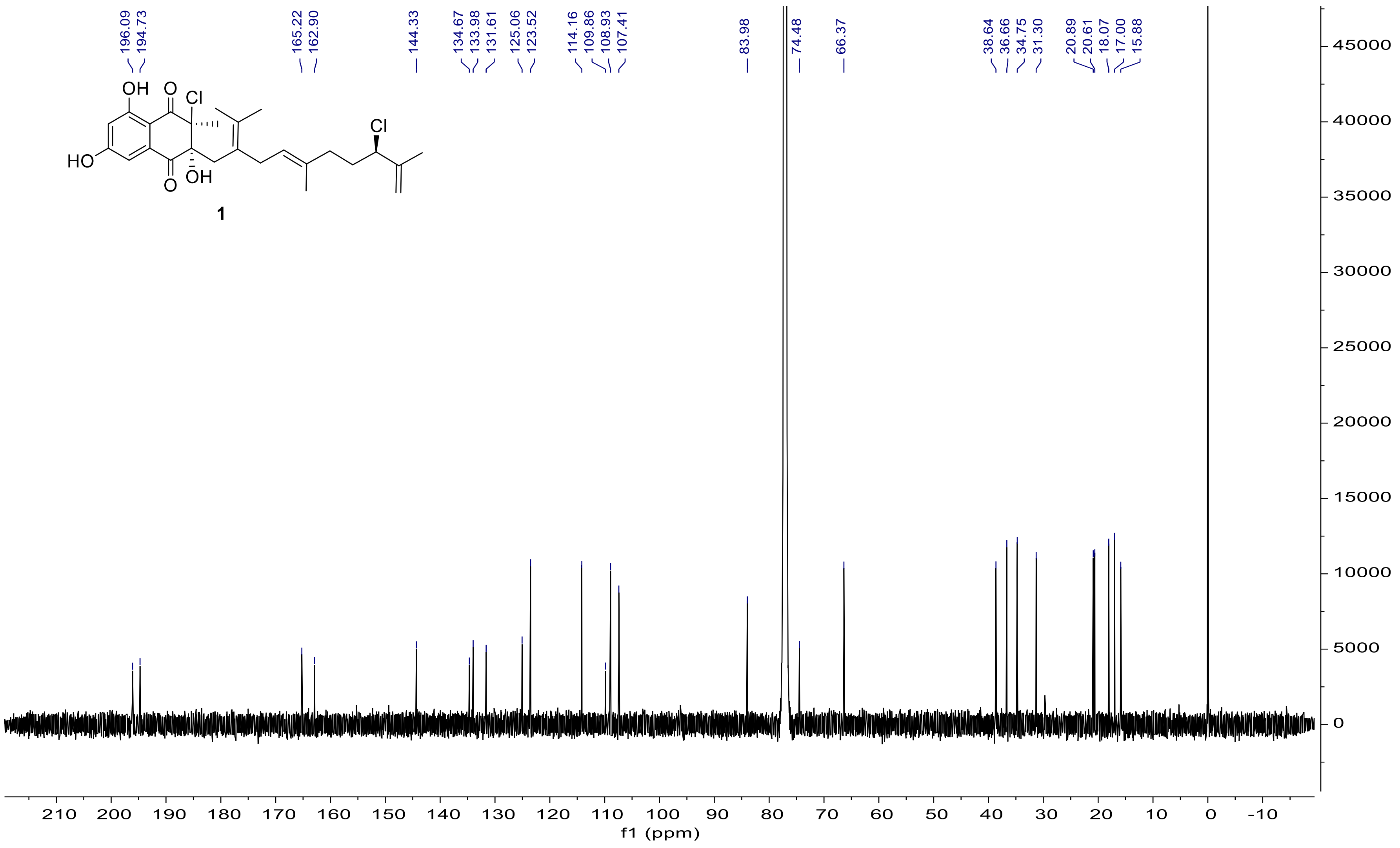

Figure S3. COSY Spectrum of Merochlorin G (**1**) in $CDCl_3$

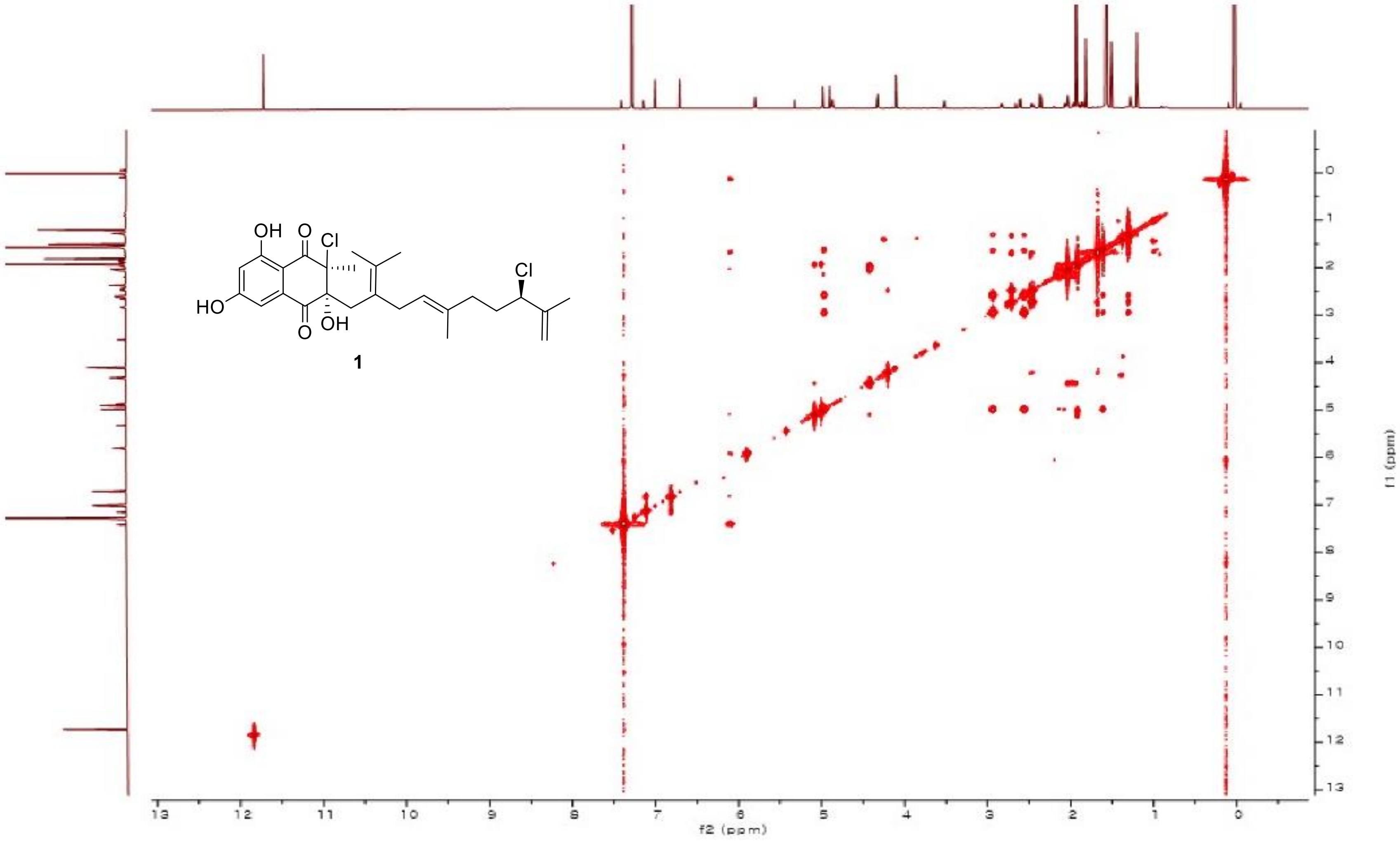

Figure S4. HSQC Spectrum of Merochlorin G (**1**) in $CDCl_3$

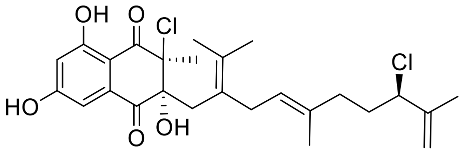

1

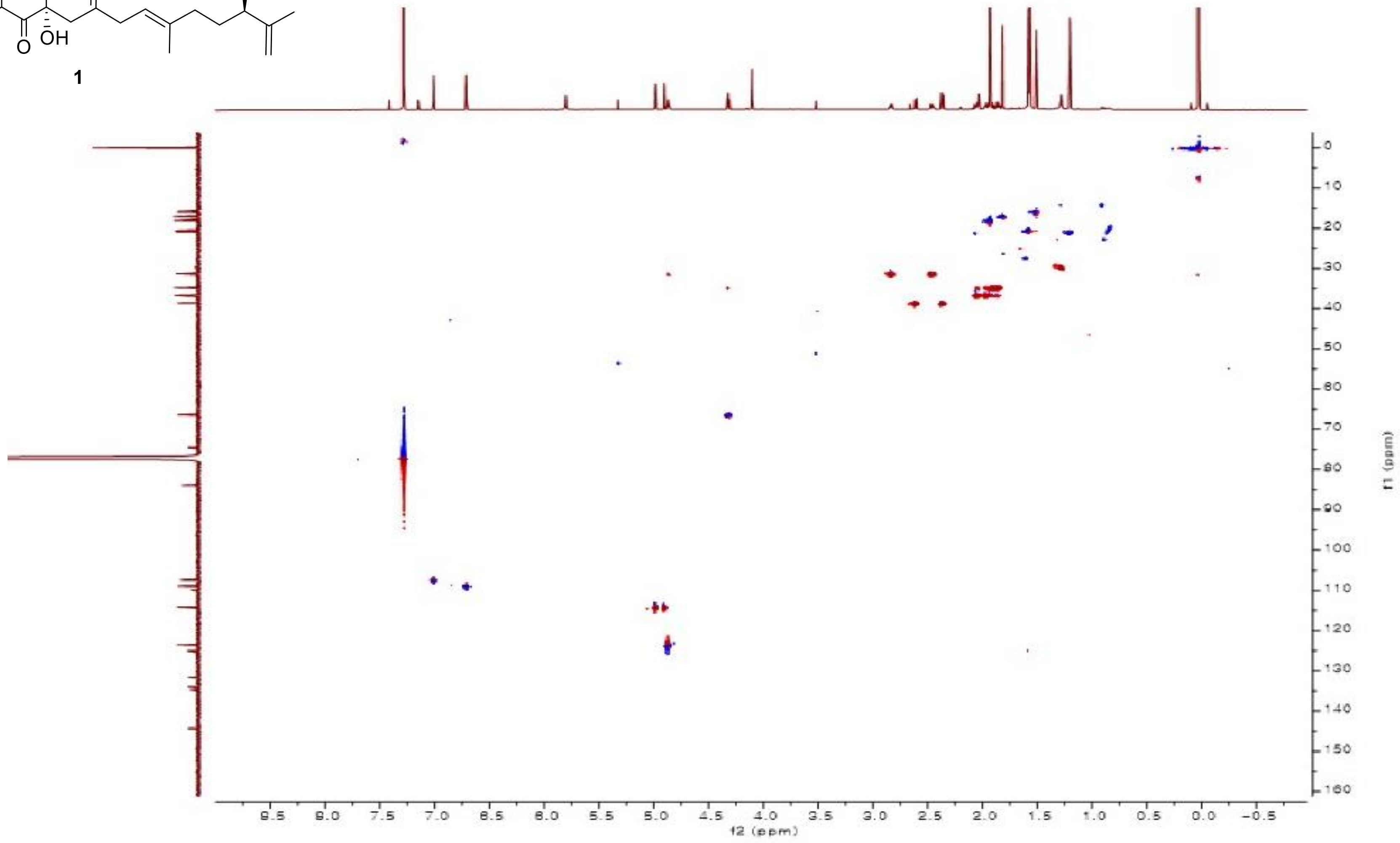

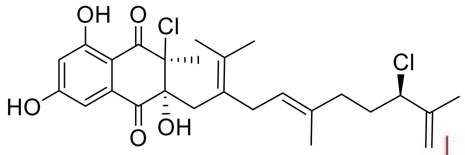

1

Figure S5. HMBC Spectrum of Merochlorin G (**1**) in $CDCl_3$

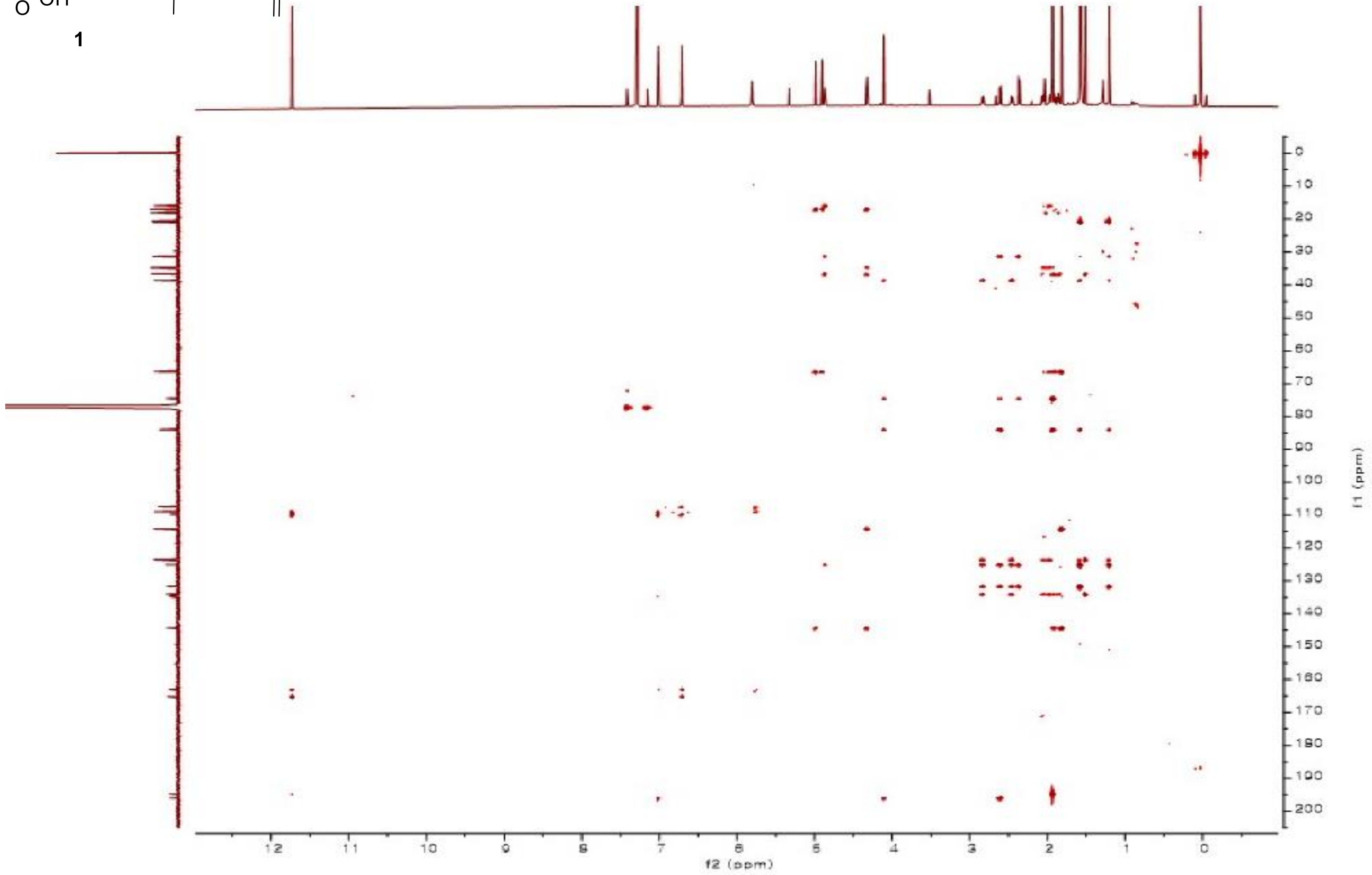

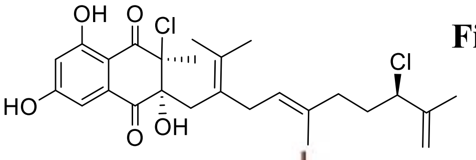

1

Figure S6. NOESY Spectrum of Merochlorin G (**1**) in $CDCl_3$

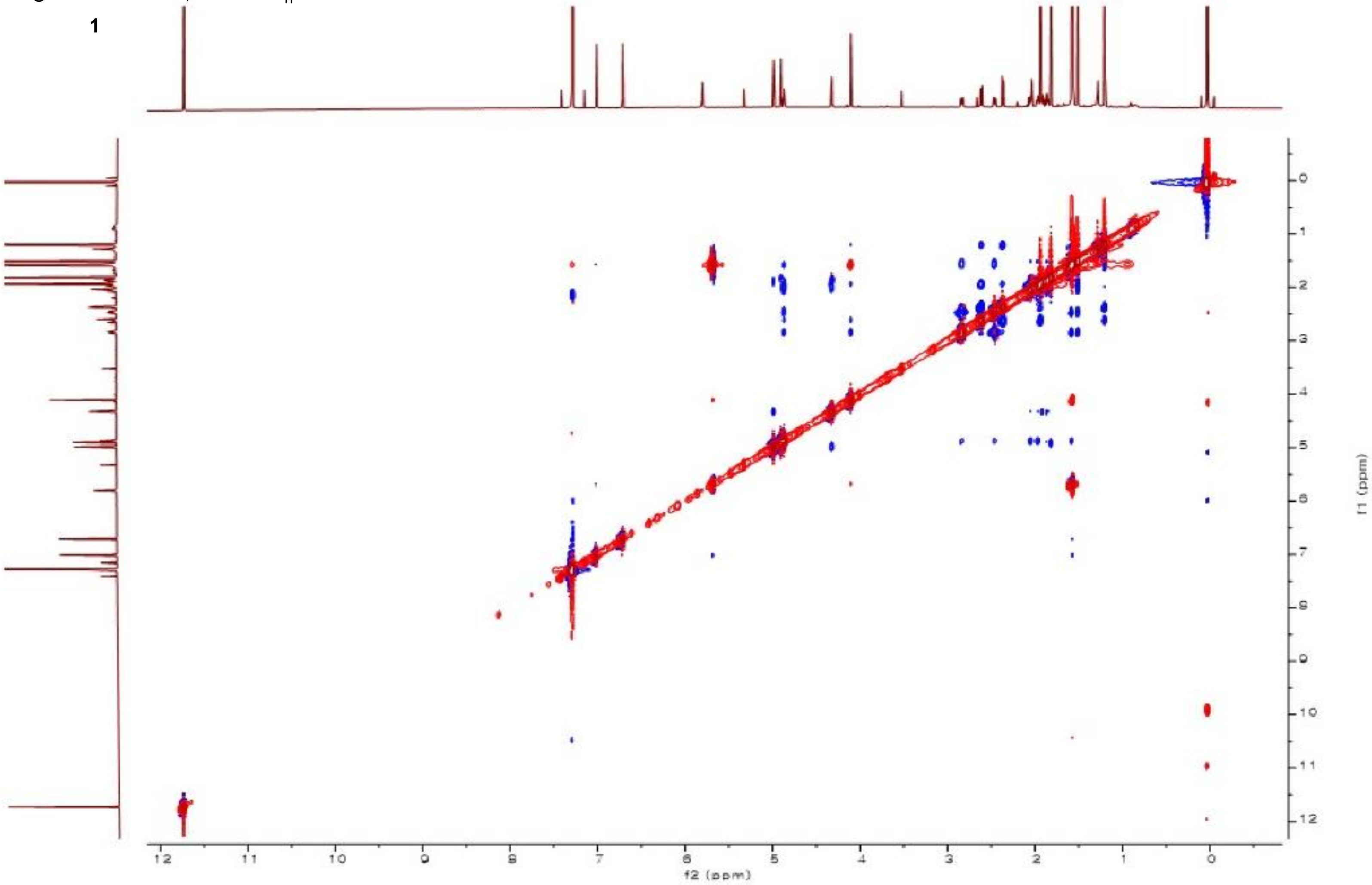

Figure S7. ^{1}H NMR Spectrum of Merochlorin H (**2**) in DMSO-d_6

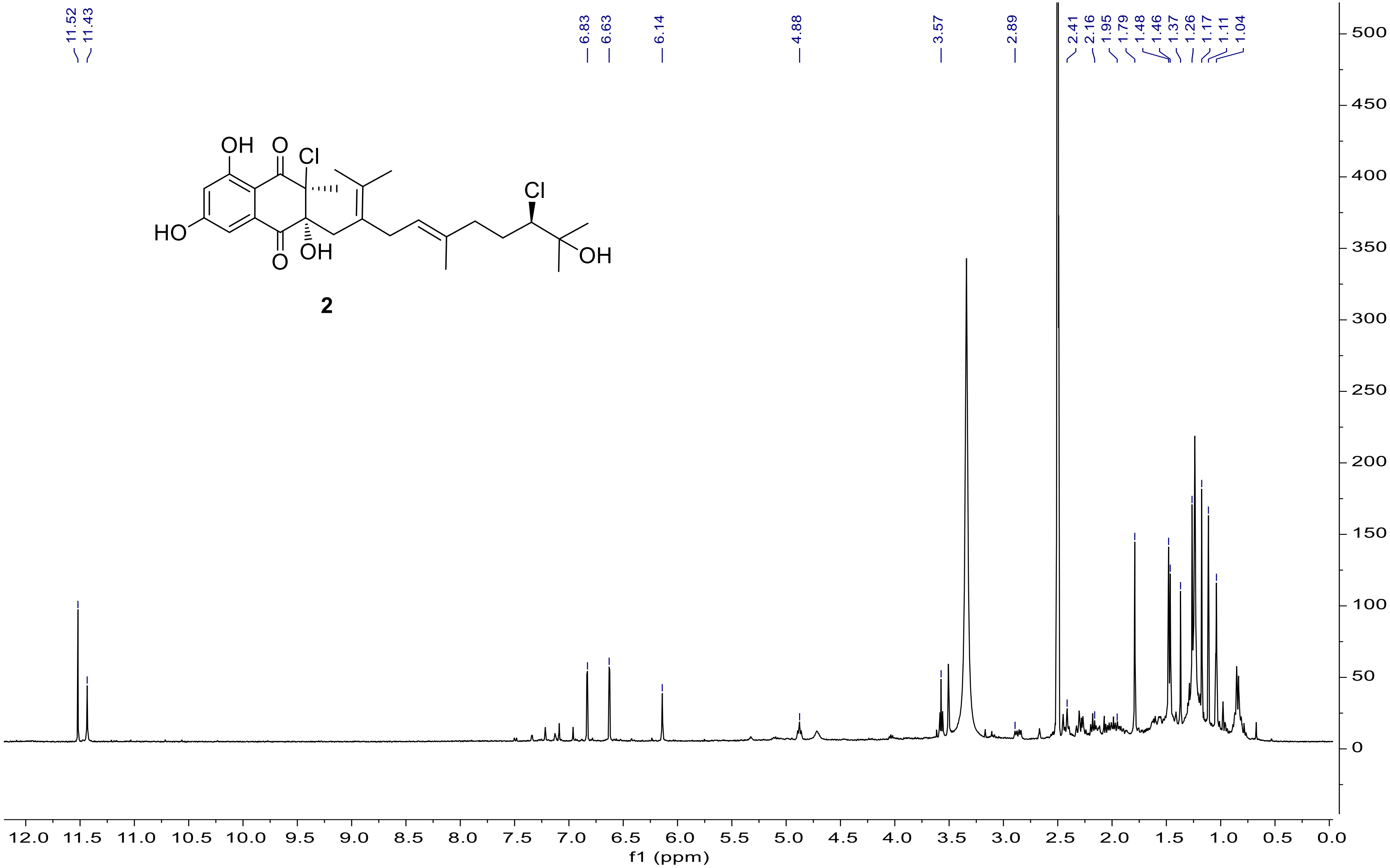

Figure S8. ^{13}C NMR Spectrum of Merochlorin H (**2**) in DMSO-d_6

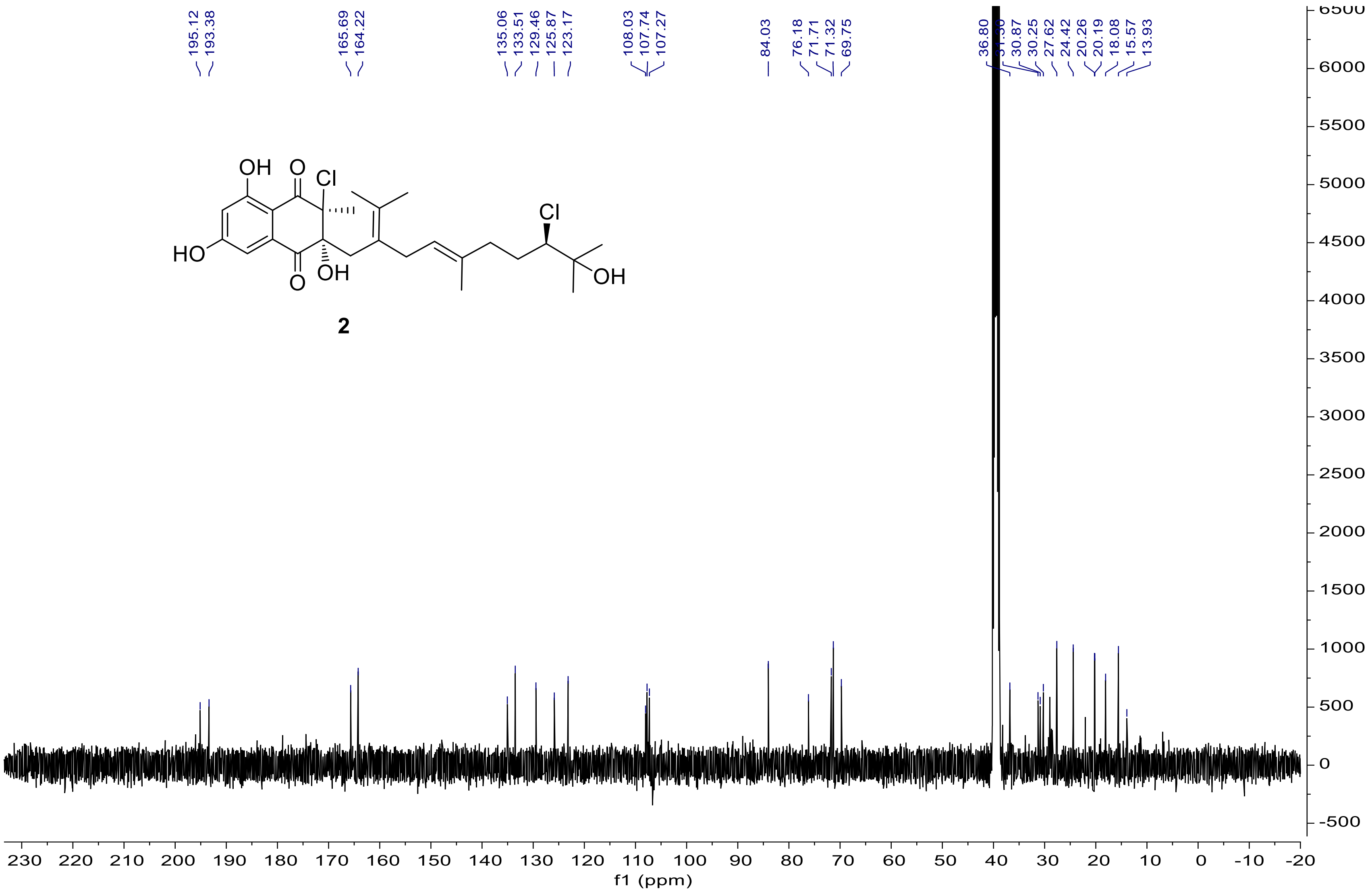

Figure S9. ^{1}H NMR Spectrum of Merochlorin I (**3**) in DMSO-d_6

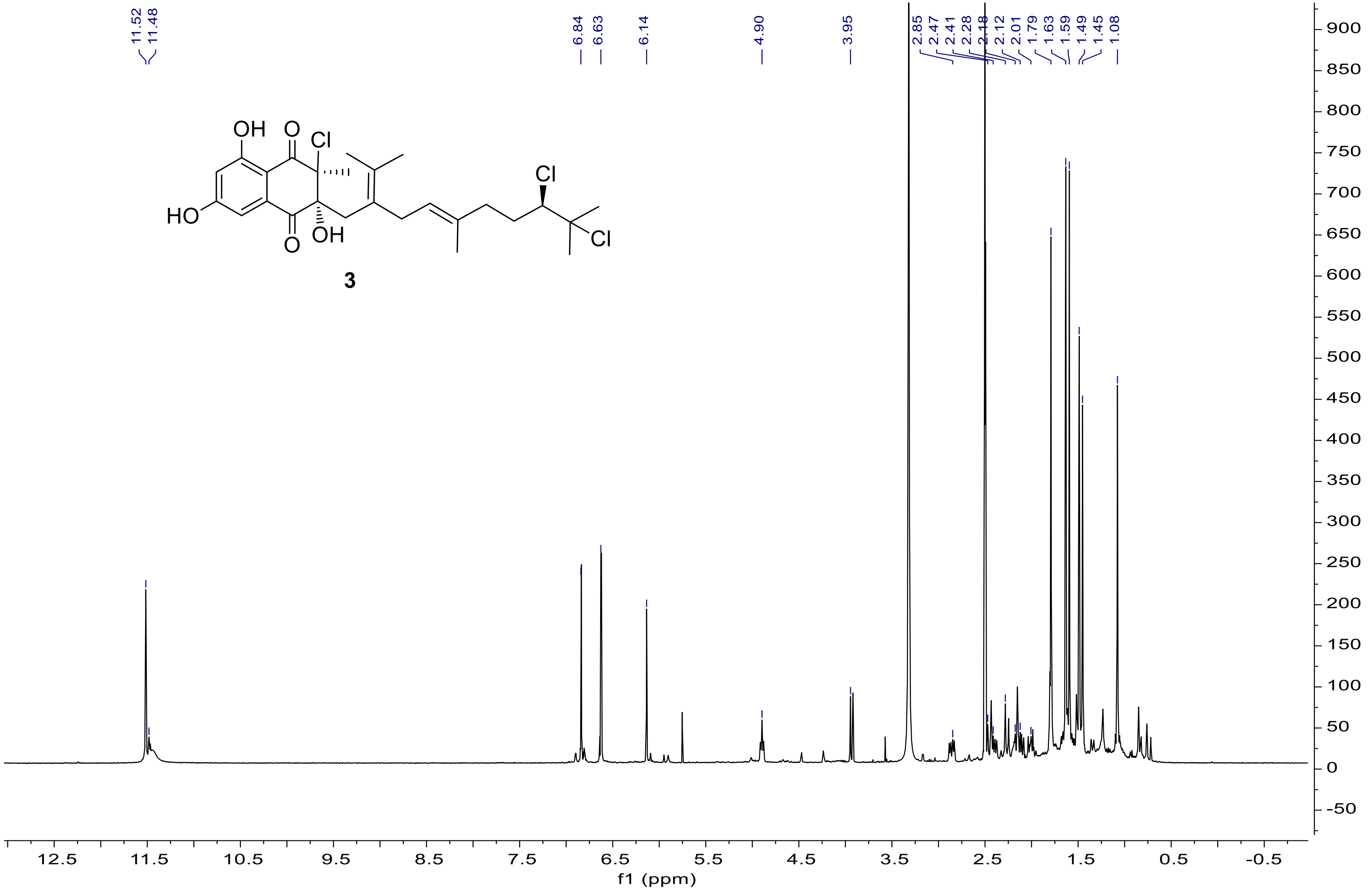

Figure S10. ^{13}C NMR Spectrum of Merochlorin I (**3**) in DMSO-d_6

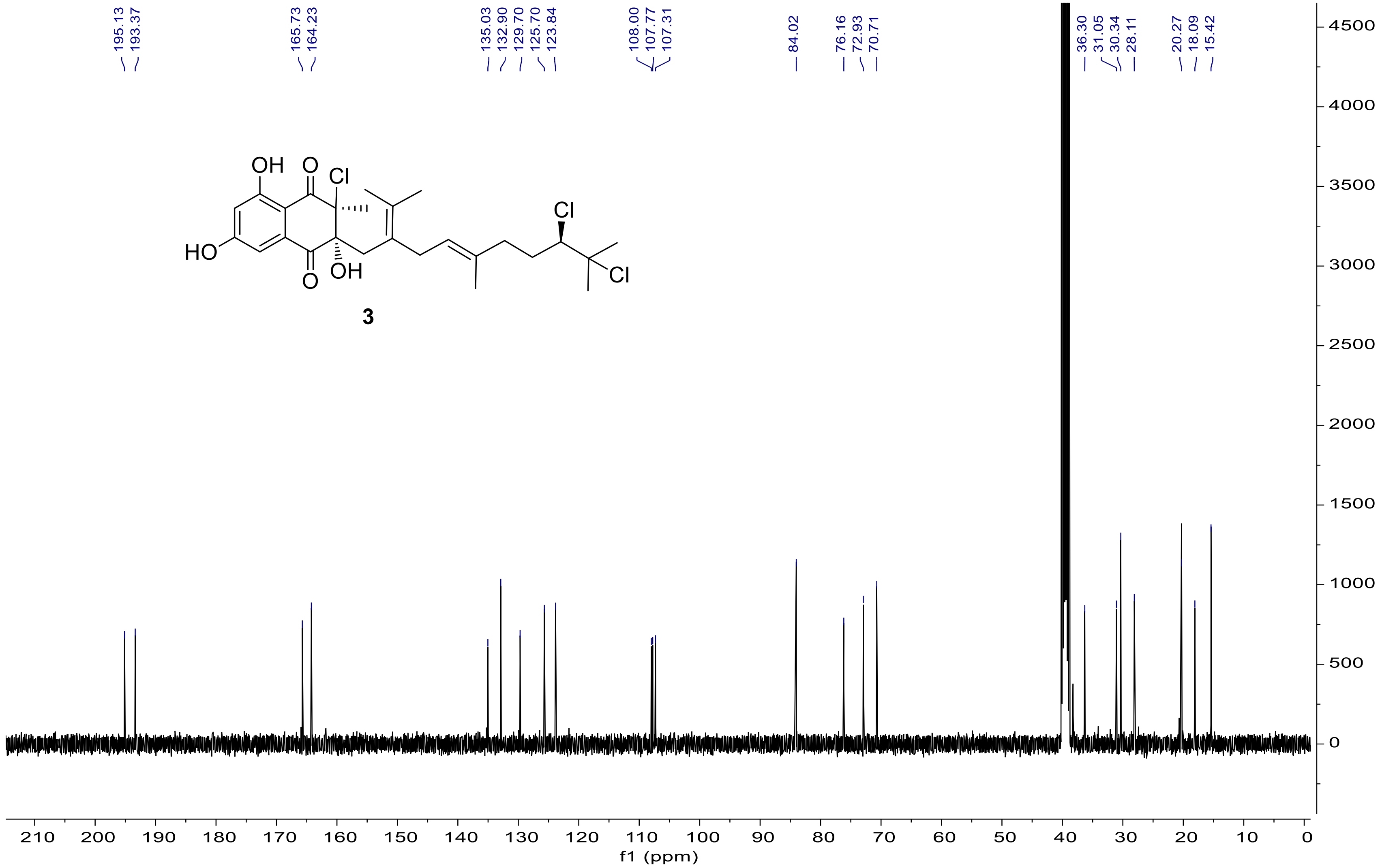

Figure S11. ^{1}H NMR Spectrum of Merochlorin J (**4**) in DMSO-d_6

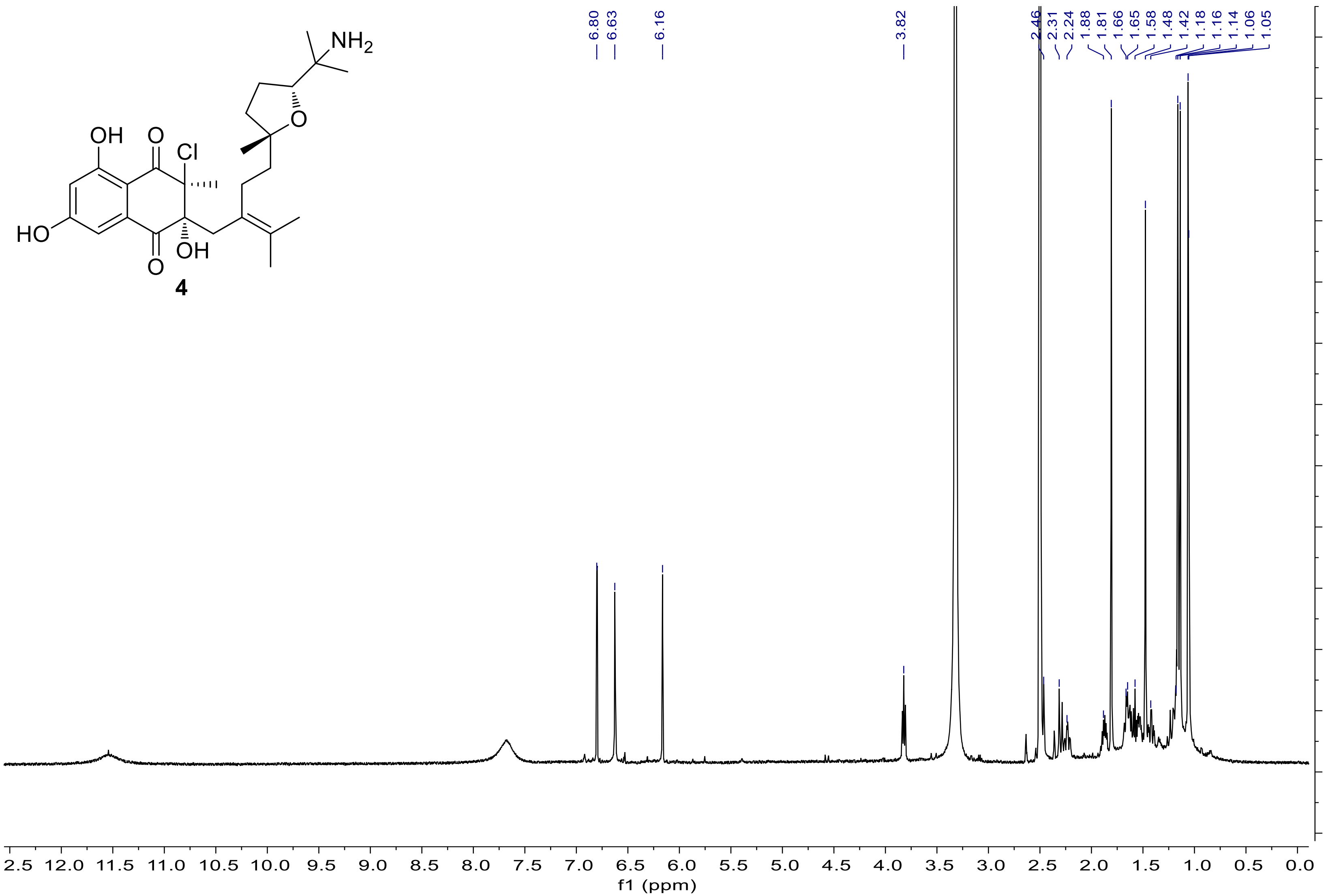

Figure S12. ^{13}C NMR Spectrum of Merochlorin J (**4**) in DMSO-d_6

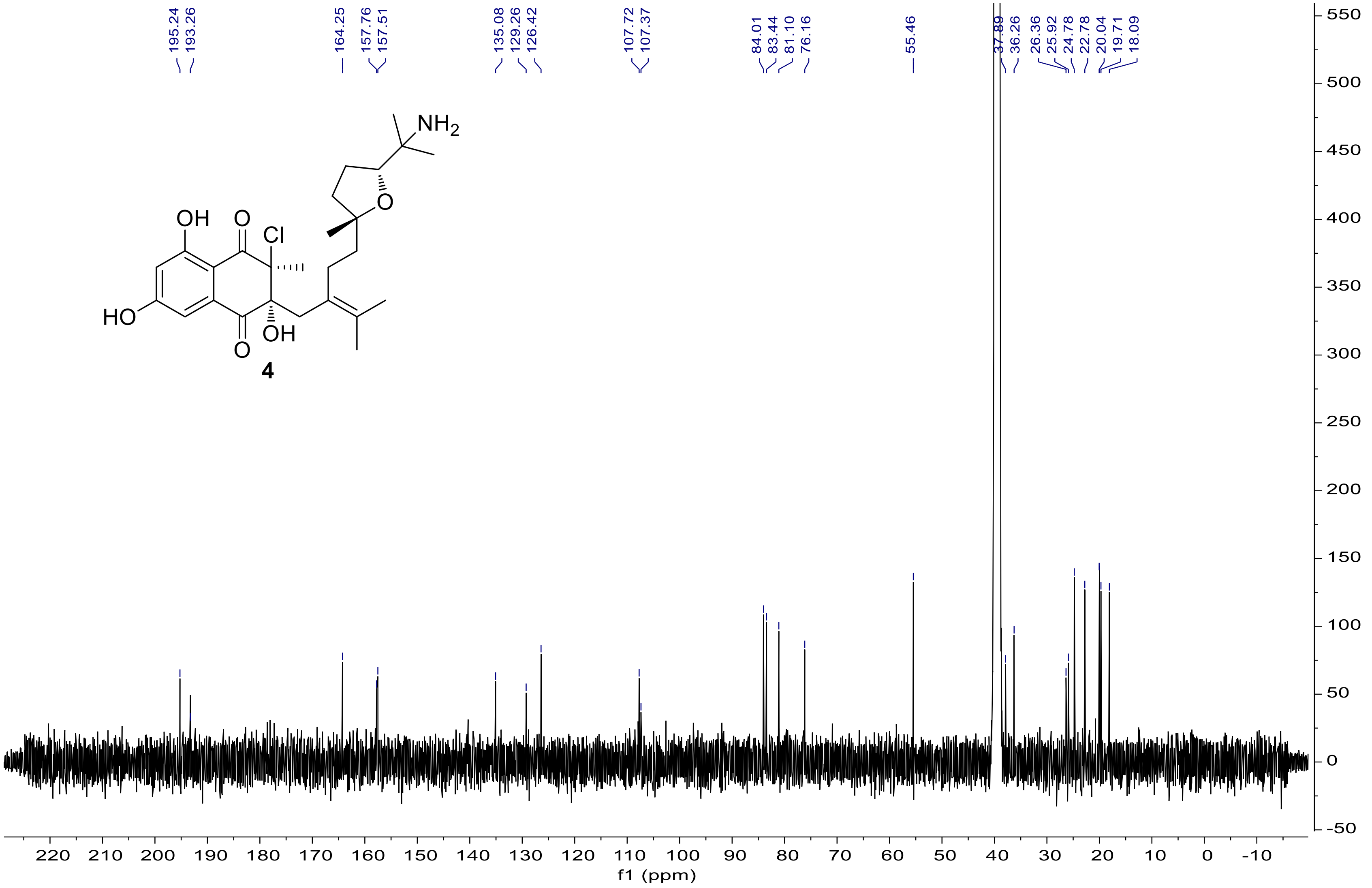

Figure S13. COSY Spectrum of Merochlorin J (**4**) in DMSO-d_6

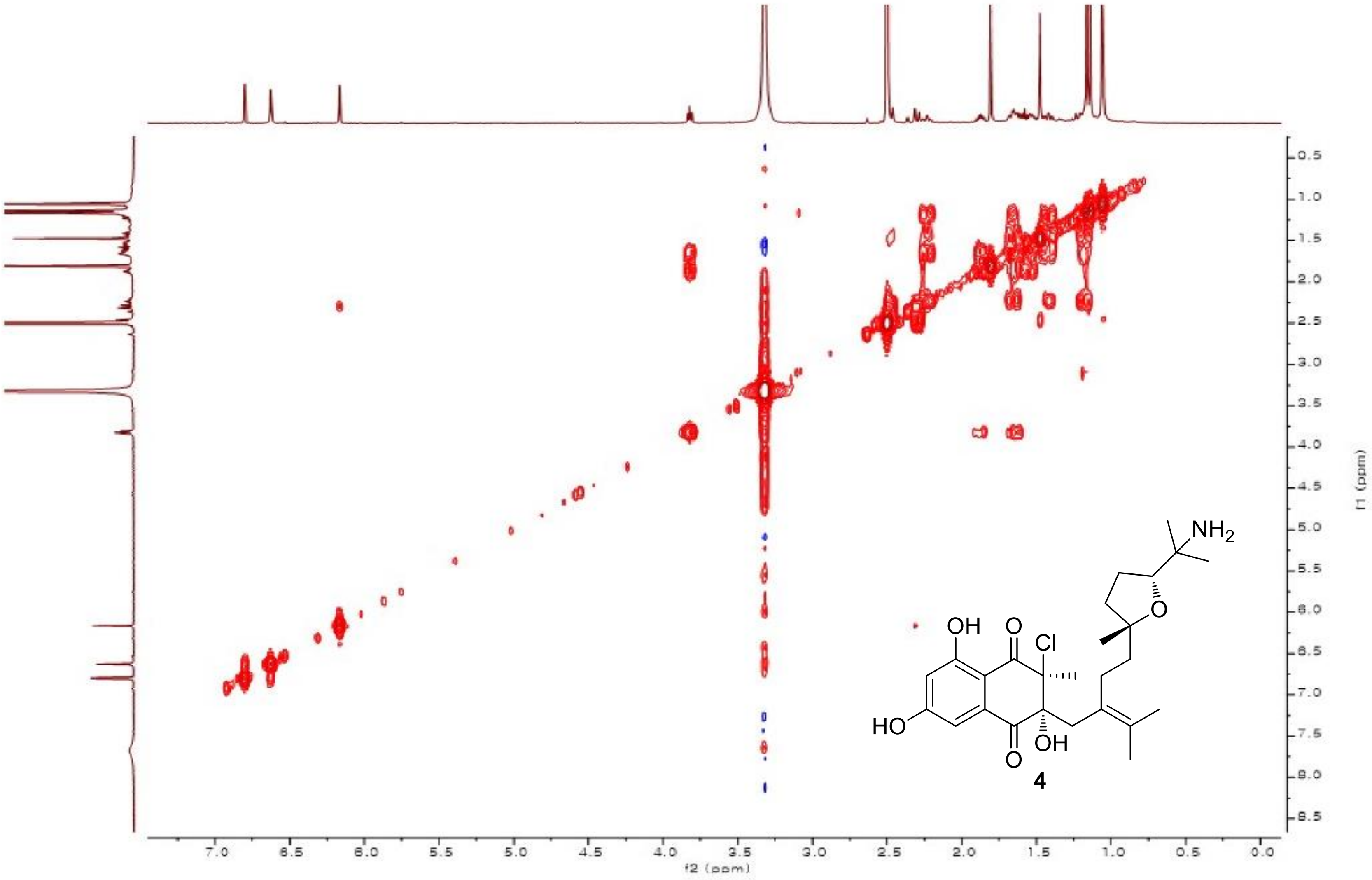

Figure S14. HSQC Spectrum of Merochlorin J (**4**) in DMSO-d_6

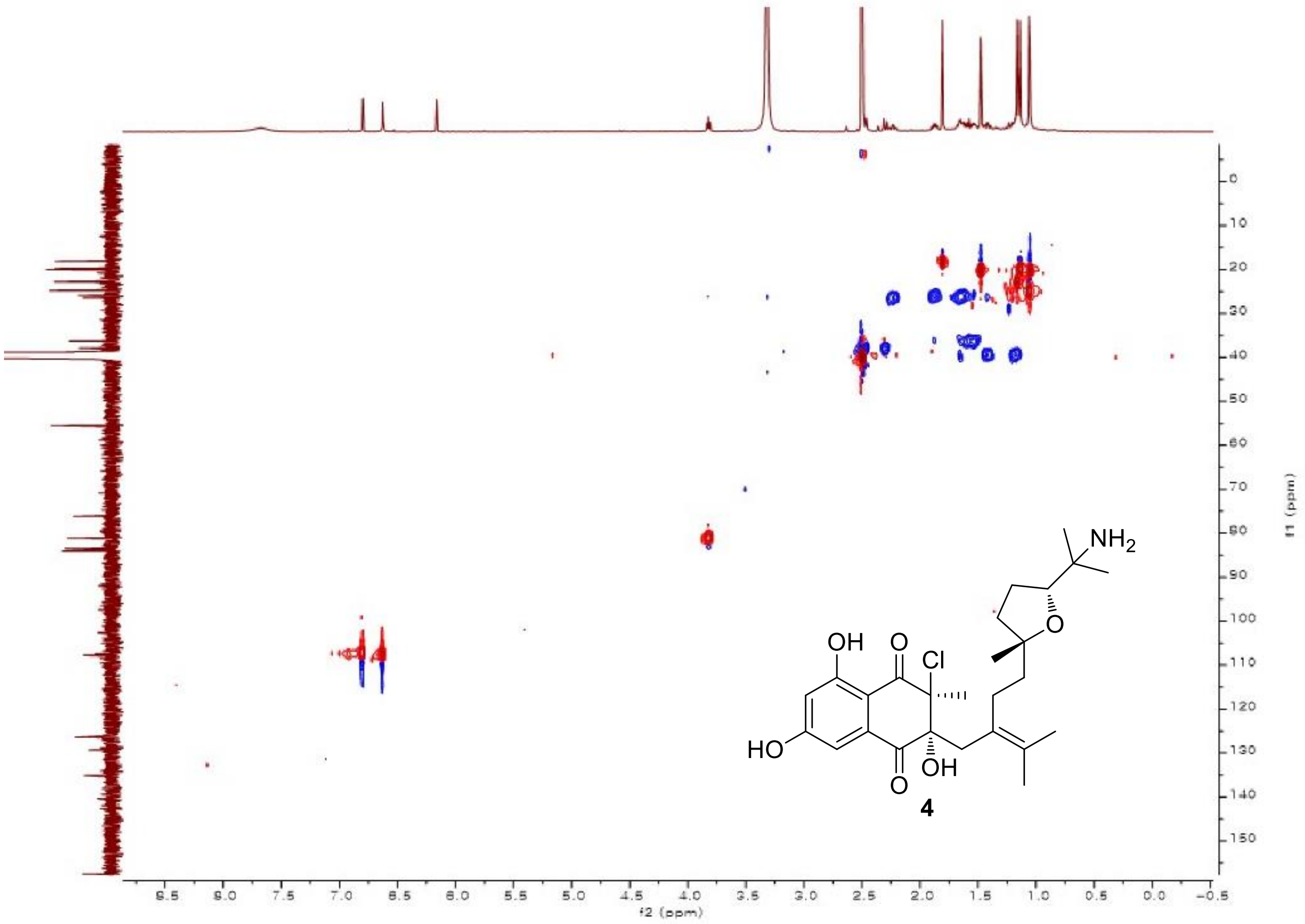

Figure S15. HMBC Spectrum of Merochlorin J (**4**) in DMSO-d_6

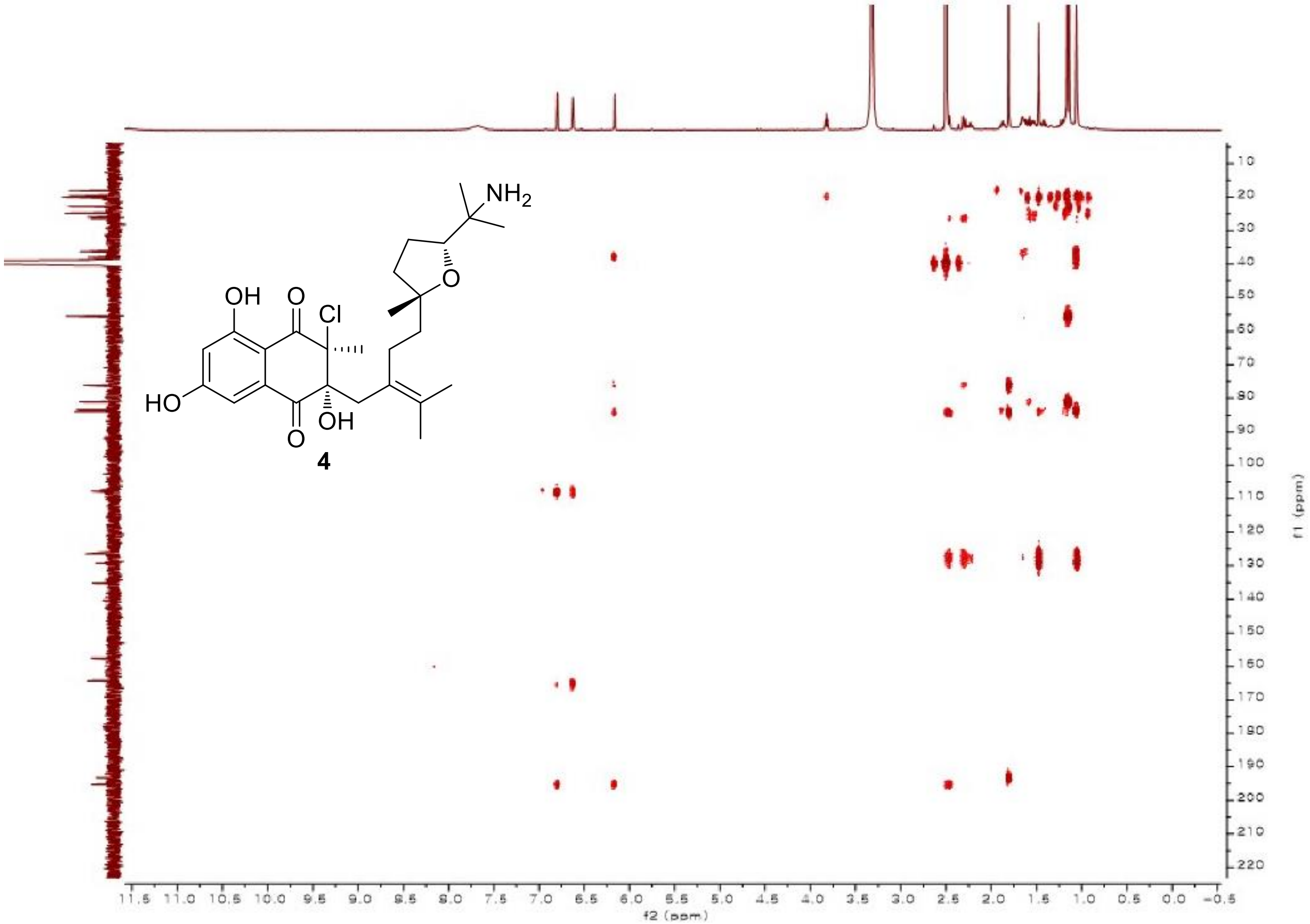

Figure S16. NOESY Spectrum of Merochlorin J (**4**) in DMSO-d_6

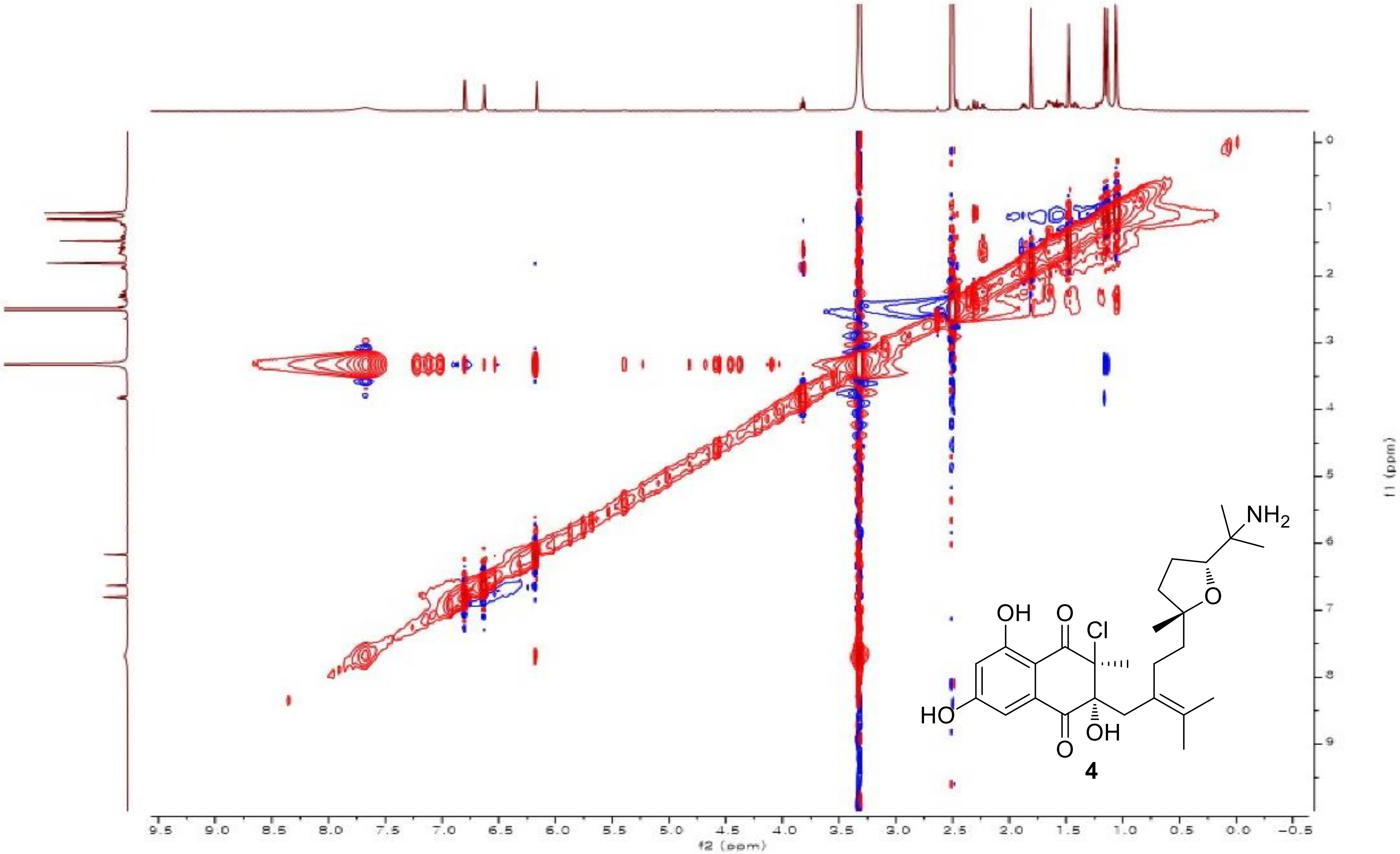

Figure S17. ^{1}H NMR Spectrum of Compound **10** in DMSO-d_6

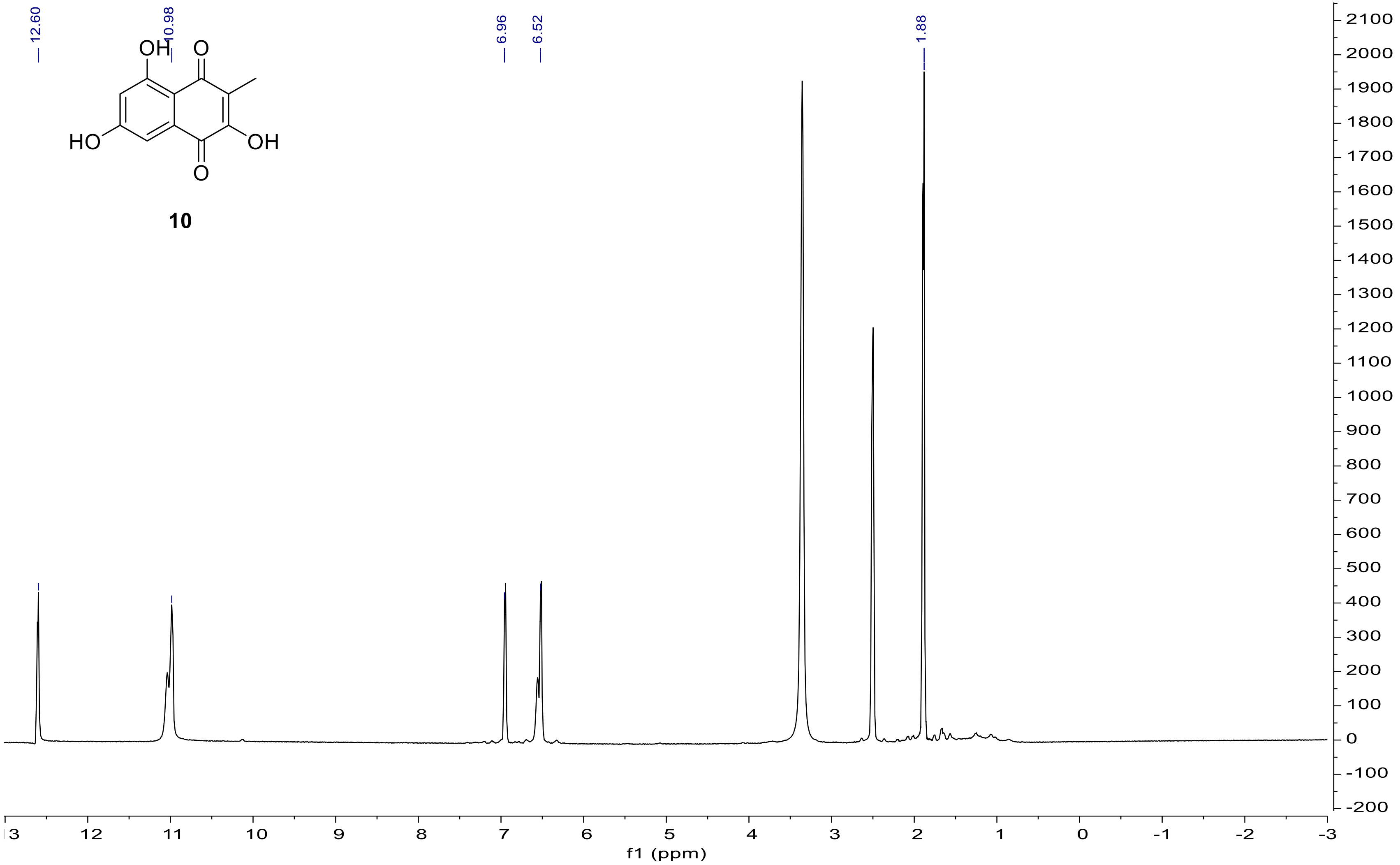

Figure S18. ^{13}C NMR Spectrum of Compound **10** in DMSO-d_6

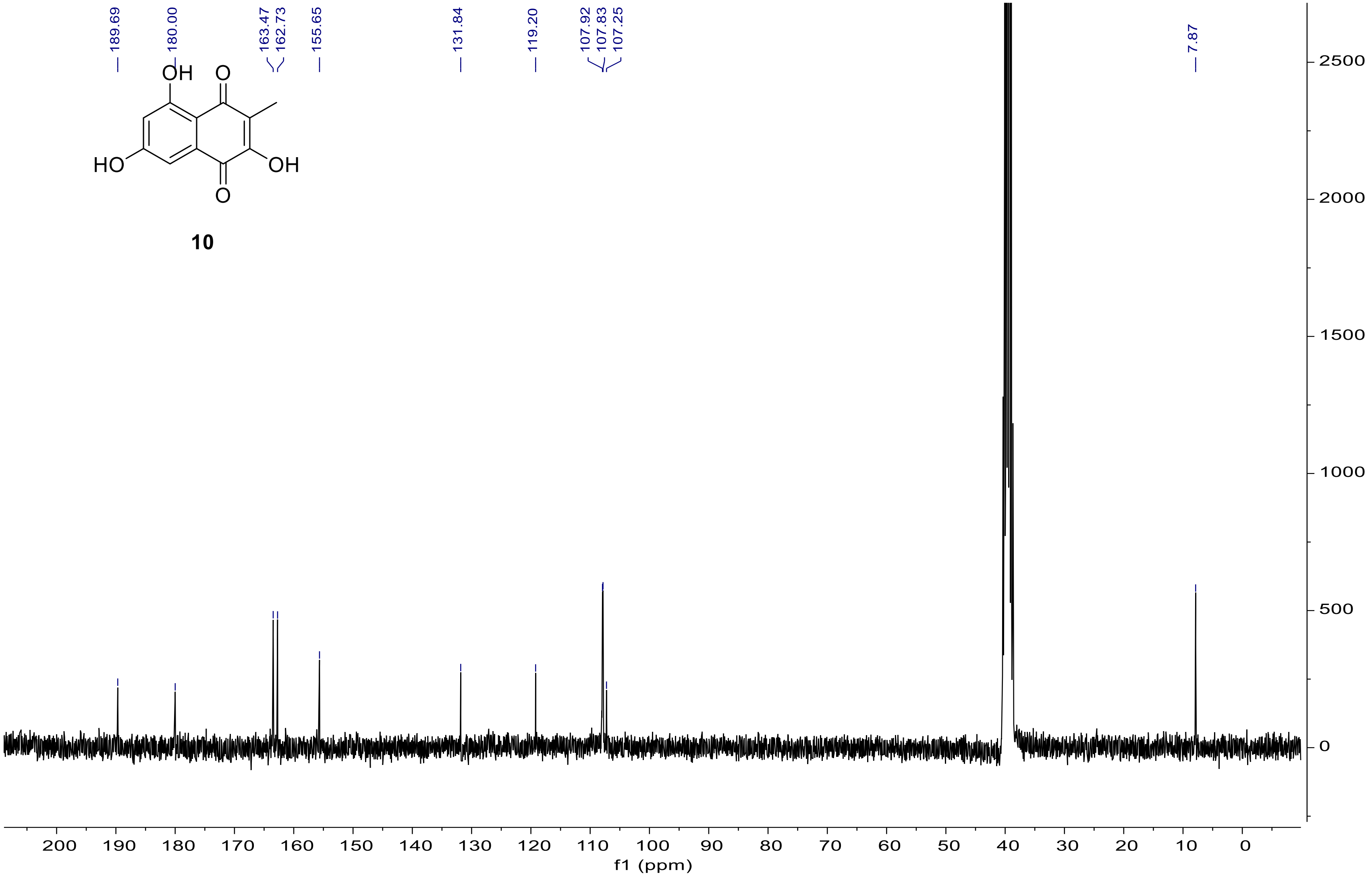

Figure S19. COSY Spectrum of Compound **10** in DMSO-d_6

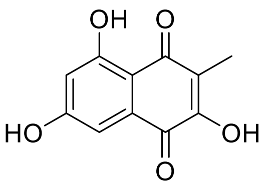

10

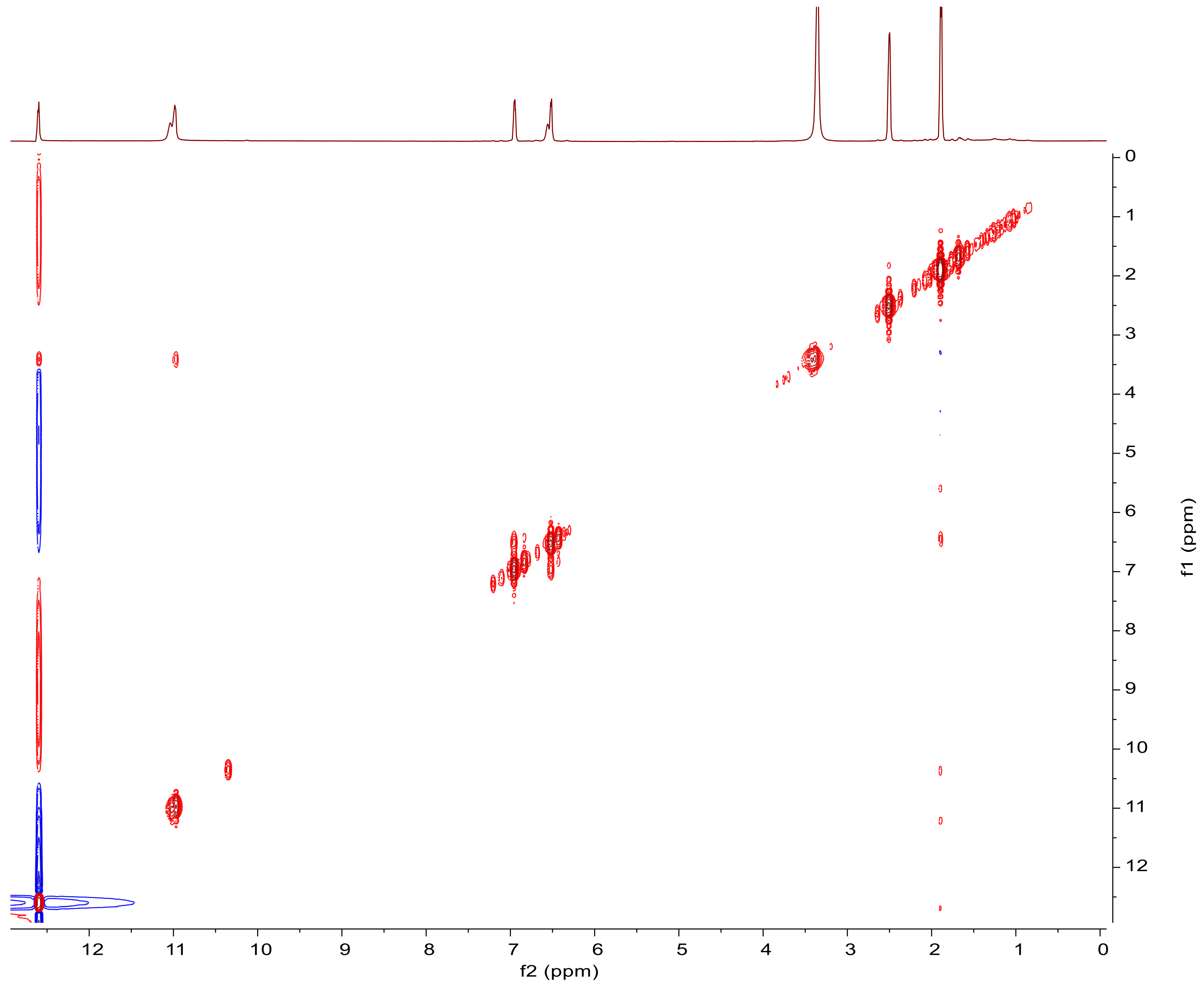

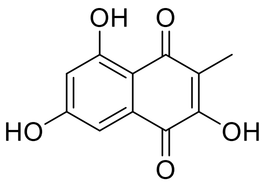

10

Figure S20. HSQC Spectrum of Compound **10** in DMSO-d_6

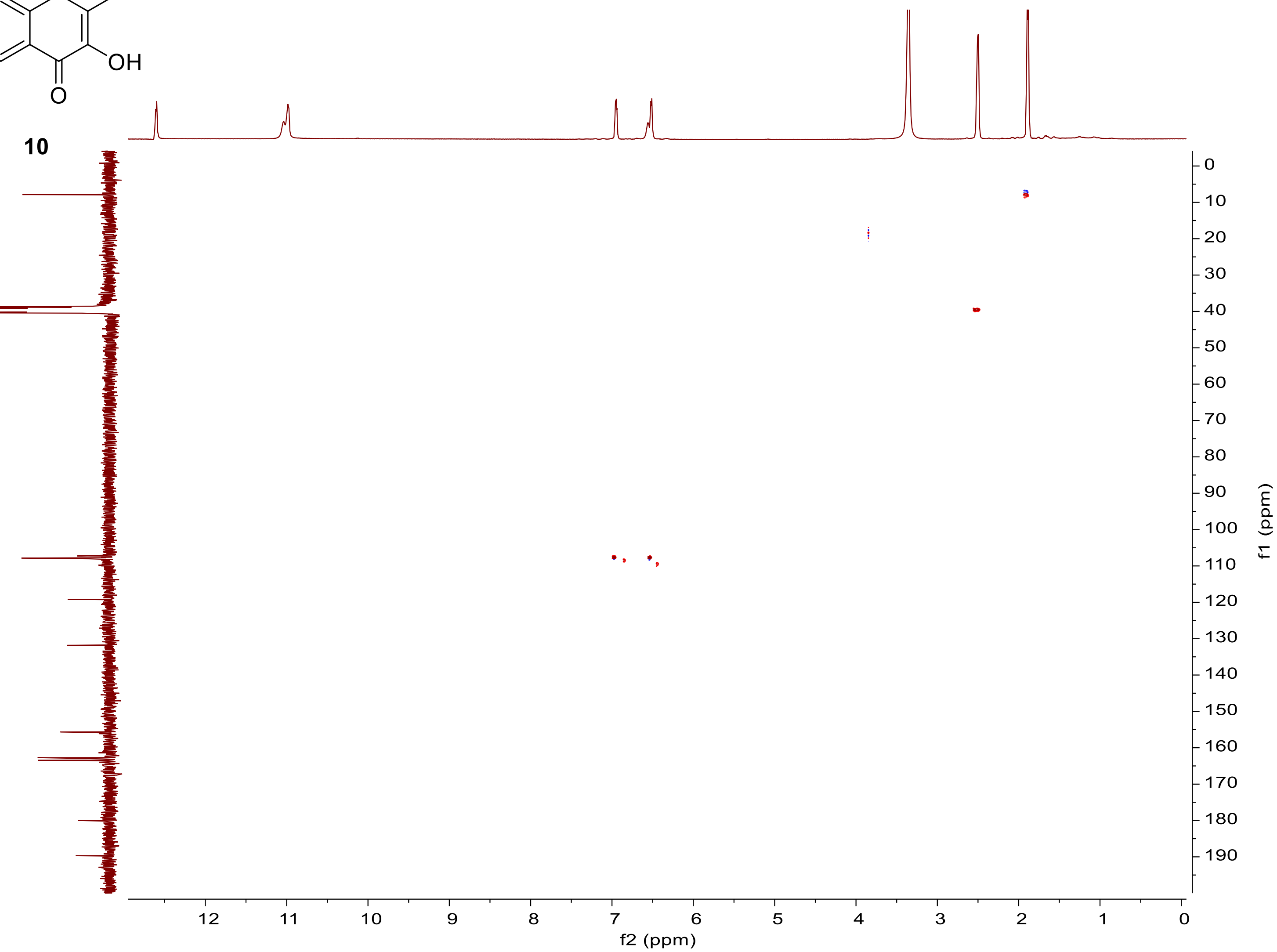

Figure S21. HMBC Spectrum of Compound **10** in DMSO-d_6

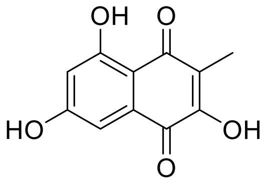

10

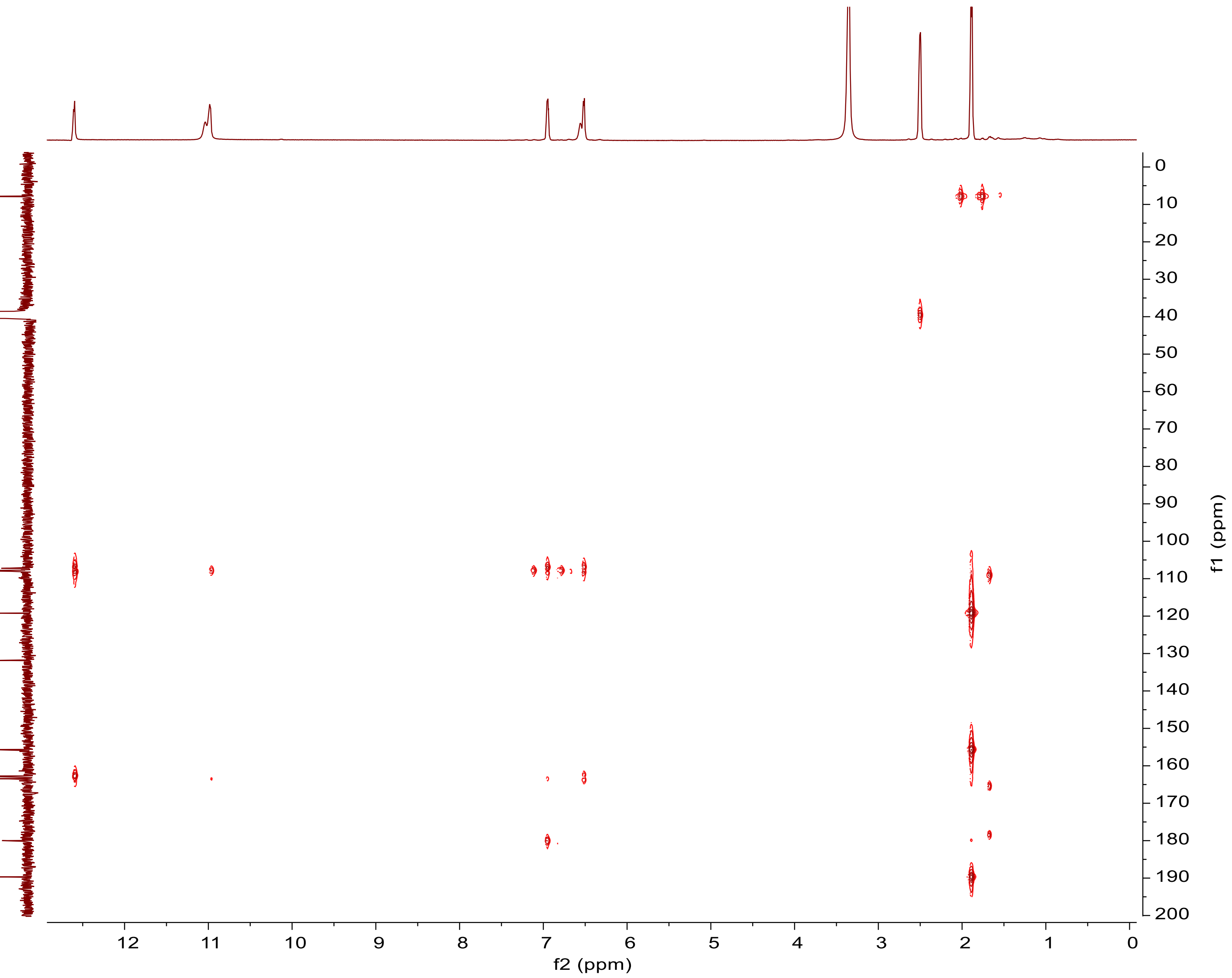

Figure S22. DP4 analysis of NMR calculation of Merochlorin G (**1**)

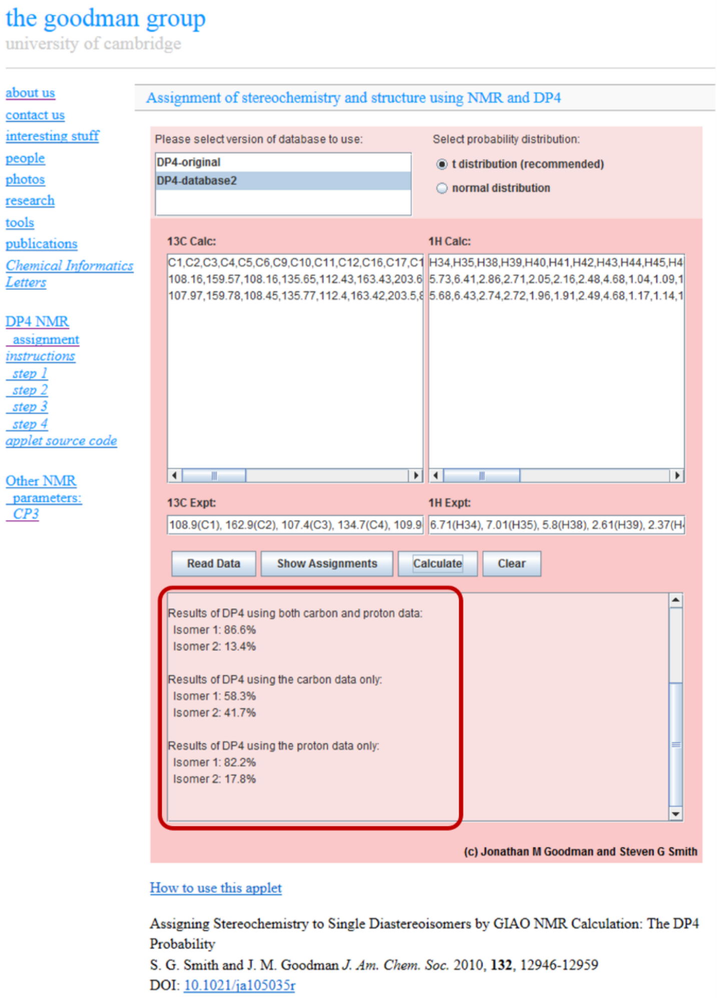

	**18*R***	**18*S***
^{13}C data	58.3%	41.7%
^{1}H data	82.2%	17.8%
Total	86.6%	13.4%

NMR data match 86.6%

18*R* configuration

Figure S23. DP4 analysis of NMR calculation of Merochlorin J (**4**)

Results of DP4 using both carbon and proton data:
Isomer 1: 100.0%
Isomer 2: 0.0%
Isomer 3: 0.0%
Isomer 4: 0.0%

Results of DP4 using the carbon data only:
Isomer 1: 0.8%
Isomer 2: 10.4%
Isomer 3: 87.7%
Isomer 4: 1.1%

Results of DP4 using the proton data only:
Isomer 1: 100.0%
Isomer 2: 0.0%
Isomer 3: 0.0%
Isomer 4: 0.0%

	**15*R*,18*R***	**15*R*,18*S***	**15*S*,18*R***	**15*S*,18*S***
^{13}C data	0.8%	10.4%	87.7%	1.1%
^{1}H data	100.0%	0.0%	0.0%	0.0%
Total	100.0%	0.0%	0.0%	0.0%

Results of DP4 using both carbon and proton data:
Isomer 1: 100.0%
Isomer 2: 0.0%
Isomer 3: 0.0%
Isomer 4: 0.0%

Results of DP4 using the carbon data only:
Isomer 1: 2.8%
Isomer 2: 69.5%
Isomer 3: 26.9%
Isomer 4: 0.8%

Results of DP4 using the proton data only:
Isomer 1: 100.0%
Isomer 2: 0.0%
Isomer 3: 0.0%
Isomer 4: 0.0%

	**15*R*,18*R***	**15*R*,18*S***	**15*S*,18*R***	**15*S*,18*S***
^{13}C data	2.8%	69.5%	26.9%	0.8%
^{1}H data	100.0%	0.0%	0.0%	0.0%
Total	100.0%	0.0%	0.0%	0.0%

Figure S24. LRMS spectrum of Merochlorins G (**1**)

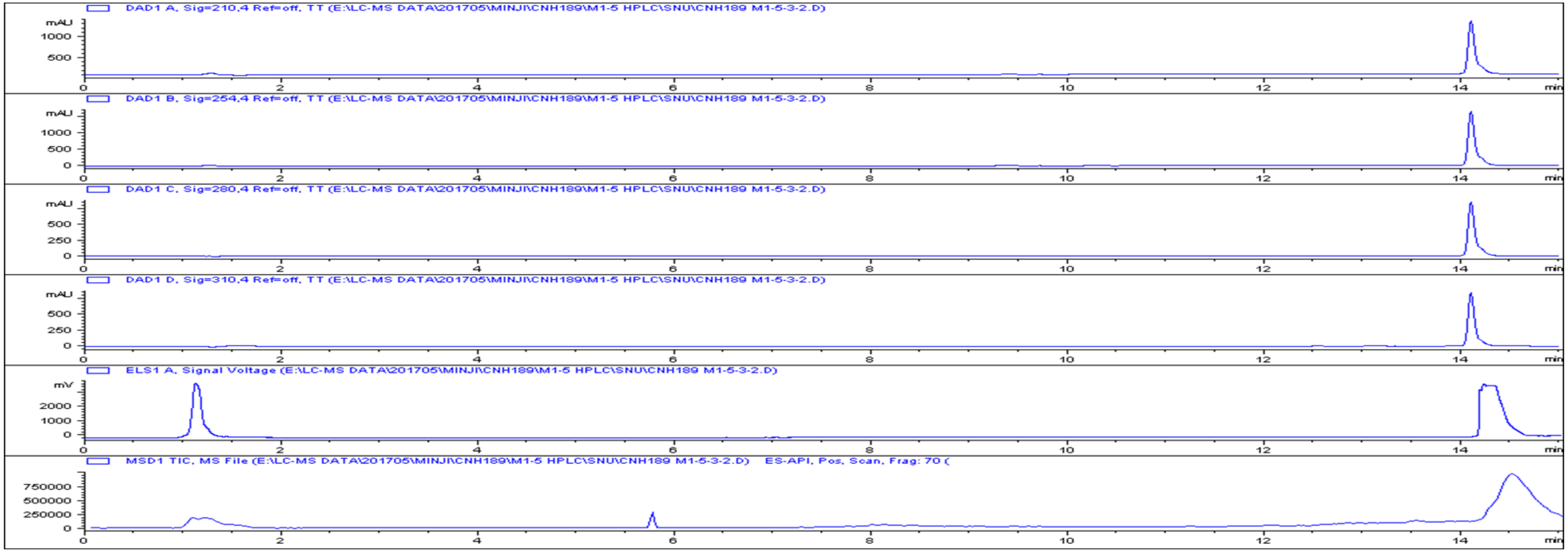

UV

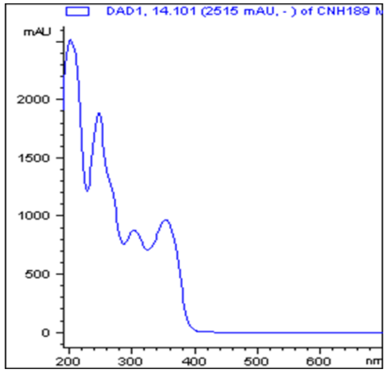

MS

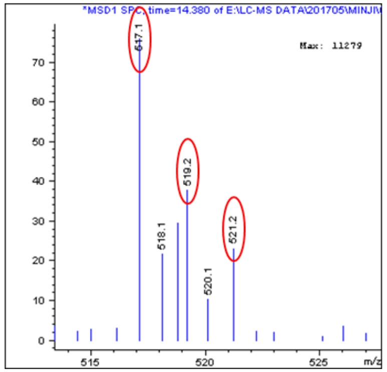

2 Cl ratio = 9:6:1

Figure S25. LRMS spectrum of Merochlorins H (**2**)

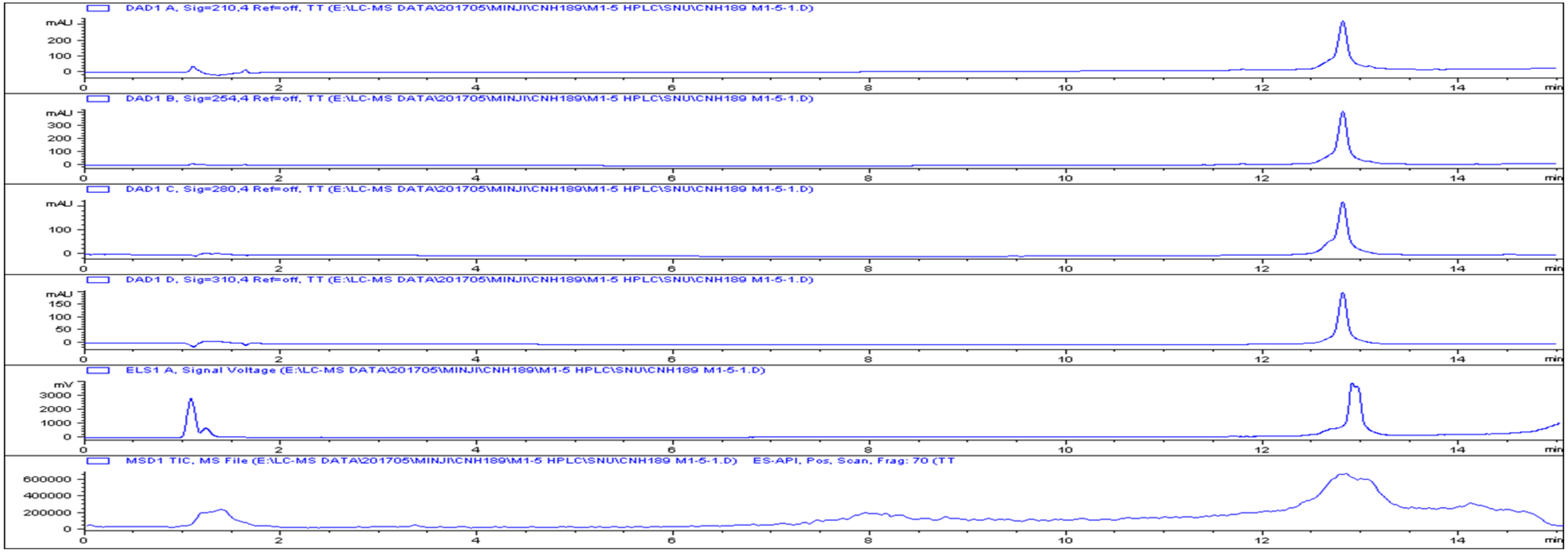

UV

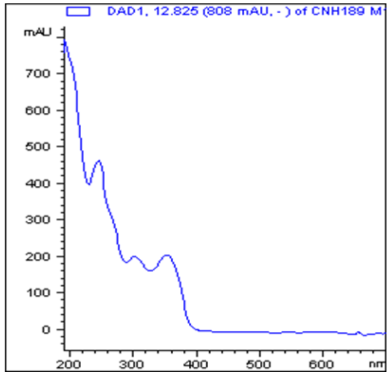

MS

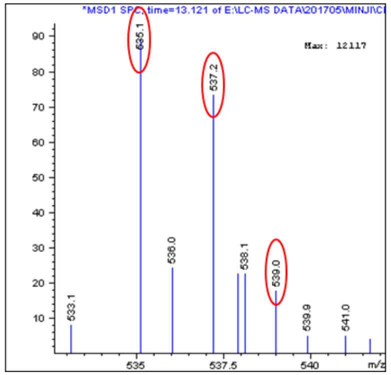

2 Cl ratio = 9:6:1

Figure S26. LRMS spectrum of Merochlorins I (**3**)

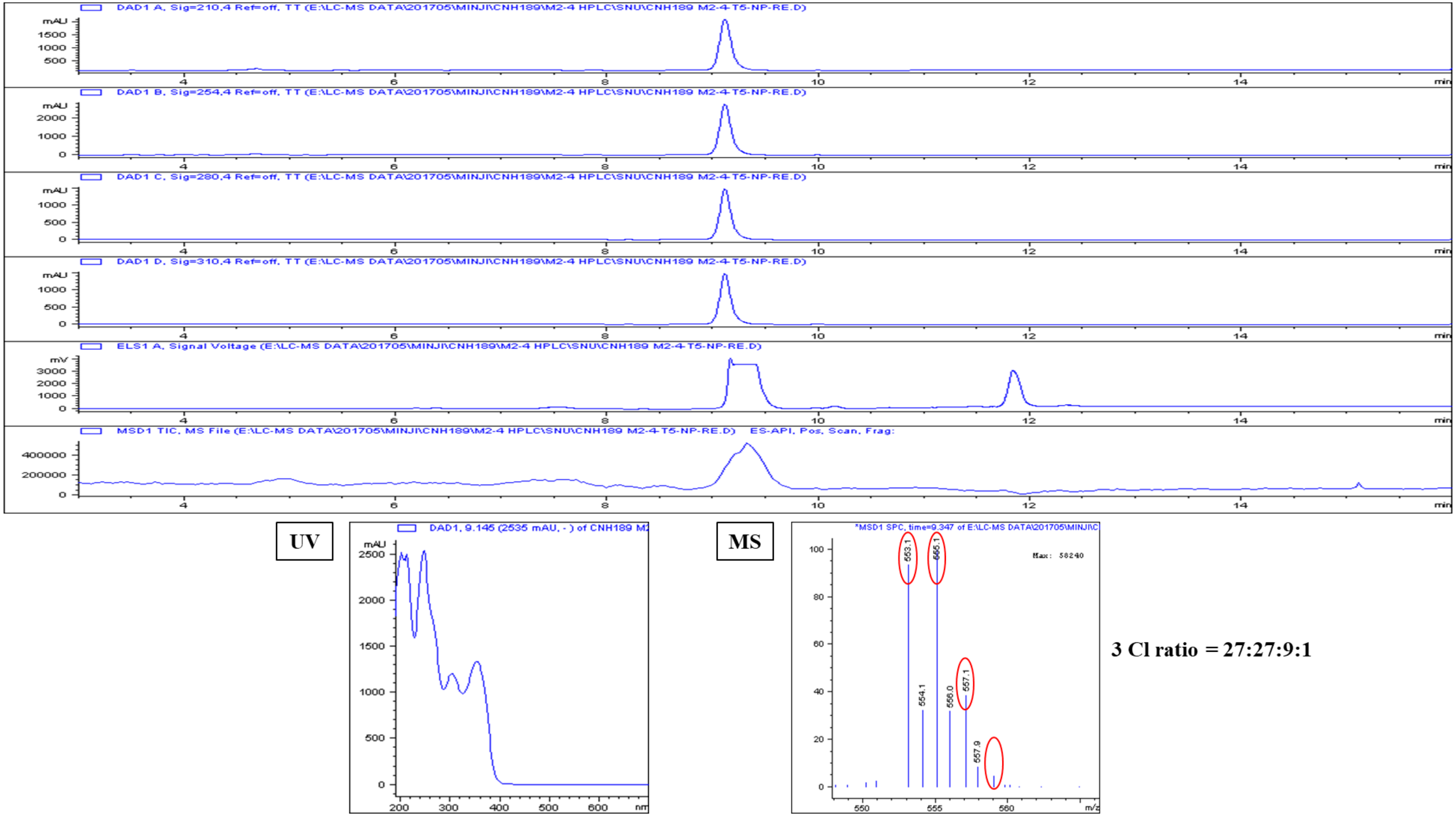

3 Cl ratio = 27:27:9:1

Figure S27. LRMS spectrum of Merochlorins J (**4**)

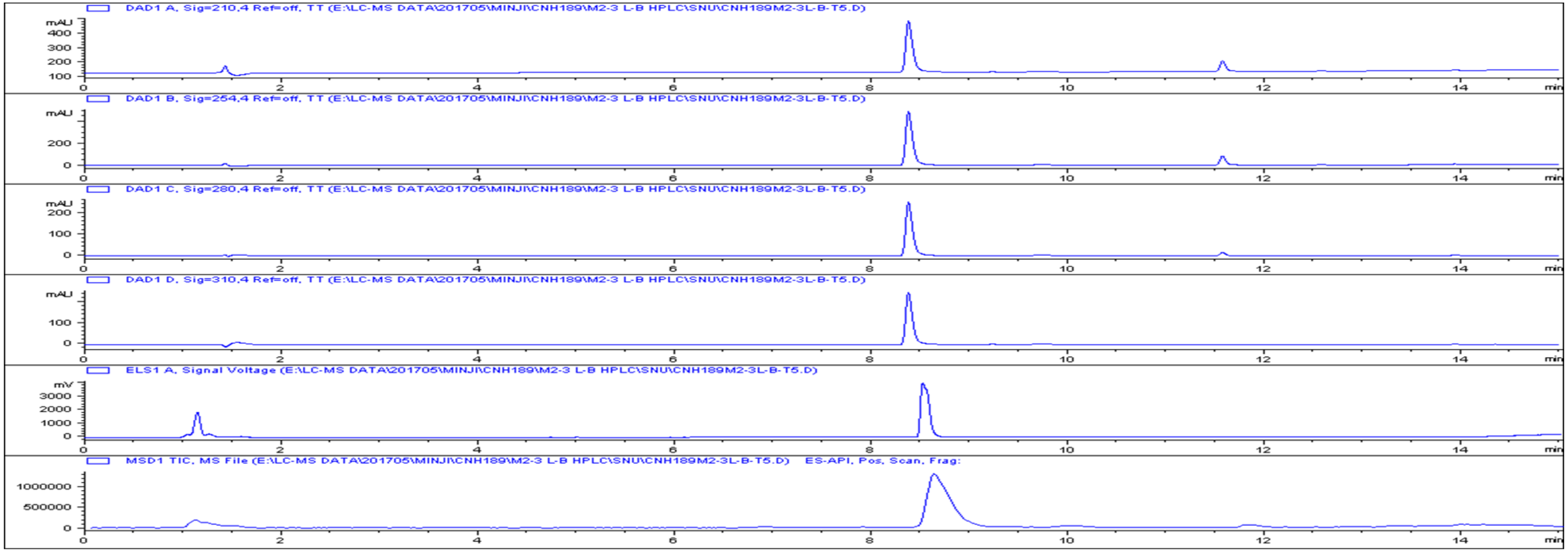

UV

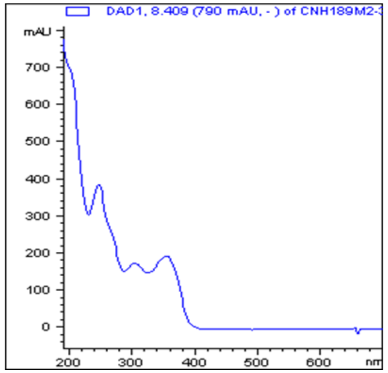

MS

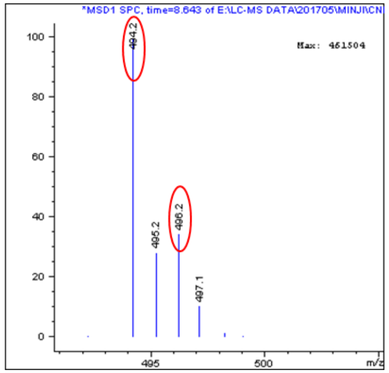

1 Cl ratio = 3:1

Figure S28. HRMS spectrum of Merochlorin G (**1**)

[Elemental Composition] Page: 1

Data : FAB-Q485 Date : 22-Feb-2018 14:22

Sample: CNH189.494B

Note : m-NBA

Inlet : Direct Ion Mode : FAB+

RT : 2.70 min Scan#: (121,130)

Elements : C 100/0, H 100/0, Cl 3/1(35Cl 3/0, 37Cl 3/0), O 10/0, Na 1/0

Mass Tolerance : 20ppm, 5mmu if m/z < 250, 10mmu if m/z > 500

Unsaturation (U.S.) : -0.5 - 30.0

Observed m/z	Int%	Err[ppm / mmu]	U.S.	Composition
517.1517	2.9	+5.3 / +2.8	21.5	C 35 H 27 35Cl 2
		-3.8 / -2.0	26.5	C 38 H 24 37Cl
		+11.1 / +5.8	13.5	C 30 H 32 35Cl 37Cl 2 O
		+9.6 / +4.9	12.5	C 29 H 32 35Cl 3 O 2
		+0.4 / +0.2	17.5	C 32 H 29 35Cl 37Cl O 2
		-10.3 / -5.3	21.5	C 34 H 26 35Cl O 3
		+6.2 / +3.2	9.5	C 27 H 34 37Cl 3 O 3
		+4.7 / +2.4	8.5	C 26 H 34 35Cl 2 37Cl O 4
		-4.5 / -2.3	13.5	C 29 H 31 37Cl 2 O 4
		-6.0 / -3.1	12.5	C 28 H 31 35Cl 2 O 5
		-15.2 / -7.8	17.5	C 31 H 28 37Cl O 5
		+19.2 / +9.9	17.5	C 30 H 26 35Cl O 6
		-0.2 / -0.1	4.5	C 23 H 36 35Cl 37Cl 2 O 6
		-1.8 / -0.9	3.5	C 22 H 36 35Cl 3 O 7
		-10.9 / -5.7	8.5	C 25 H 33 35Cl 37Cl O 7
		+14.3 / +7.4	13.5	C 27 H 28 37Cl O 8
		-5.1 / -2.6	0.5	C 20 H 38 37Cl 3 O 8
		-6.7 / -3.5	-0.5	C 19 H 38 35Cl 2 37Cl O 9
		-15.8 / -8.2	4.5	C 22 H 35 37Cl 2 O 9
		-17.4 / -9.0	3.5	C 21 H 35 35Cl 2 O 10
		+18.6 / +9.6	4.5	C 21 H 33 35Cl 37Cl O 10
		+10.0 / +5.2	18.5	C 33 H 28 35Cl 2 Na
		+0.8 / +0.4	23.5	C 36 H 25 37Cl Na
		-18.6 / -9.6	10.5	C 29 H 35 37Cl 3 Na
		+15.8 / +8.2	10.5	C 28 H 33 35Cl 37Cl 2 O Na
		+14.2 / +7.4	9.5	C 27 H 33 35Cl 3 O 2 Na
		+5.1 / +2.6	14.5	C 30 H 30 35Cl 37Cl O 2 Na
		-5.6 / -2.9	18.5	C 32 H 27 35Cl O 3 Na
		+10.9 / +5.6	6.5	C 25 H 35 37Cl 3 O 3 Na
		+9.3 / +4.8	5.5	C 24 H 35 35Cl 2 37Cl O 4 Na
		+0.2 / +0.1	10.5	C 27 H 32 37Cl 2 O 4 Na
		-1.4 / -0.7	9.5	C 26 H 32 35Cl 2 O 5 Na
		-10.5 / -5.4	14.5	C 29 H 29 37Cl O 5 Na
		+4.4 / +2.3	1.5	C 21 H 37 35Cl 37Cl 2 O 6 Na
		+2.9 / +1.5	0.5	C 20 H 37 35Cl 3 O 7 Na
		-6.3 / -3.2	5.5	C 23 H 34 35Cl 37Cl O 7 Na
		-17.0 / -8.8	9.5	C 25 H 31 35Cl O 8 Na
		+19.0 / +9.8	10.5	C 25 H 29 37Cl O 8 Na
		-11.2 / -5.8	1.5	C 20 H 36 37Cl 2 O 9 Na
		-12.7 / -6.6	0.5	C 19 H 36 35Cl 2 O 10 Na

Figure S29. HRMS spectrum of Merochlorin H (**2**)

[Elemental Composition]   Page: 1
Data   : FAB-Q484   Date : 22-Feb-2018 14:08
Sample: CNH189.512
Note : m-NBA
Inlet : Direct   Ion Mode : FAB+
RT : 5.13 min   Scan#: (222,254)
Elements : C 100/0, H 100/0, Cl 3/1(35Cl 3/0, 37Cl 3/0), O 10/0, Na 1/0
Mass Tolerance   : 20ppm, 5mmu if m/z < 250, 10mmu if m/z > 500
Unsaturation (U.S.) : -0.5 - 30.0

Observed m/z	Int%	Err[ppm / mmu]	U.S.	Composition
535.1627	4.1	-18.6 / -10.0	15.5	C 33 H 34 35Cl 3
		+16.2 / +8.6	16.5	C 33 H 32 35Cl 2 37Cl
		+7.3 / +3.9	21.5	C 36 H 29 37Cl 2
		+5.8 / +3.1	20.5	C 35 H 29 35Cl 2 O
		-3.0 / -1.6	25.5	C 38 H 26 37Cl O
		+11.4 / +6.1	12.5	C 30 H 34 35Cl 37Cl 2 O 2
		+9.9 / +5.3	11.5	C 29 H 34 35Cl 3 O 3
		+1.1 / +0.6	16.5	C 32 H 31 35Cl 37Cl O 3
		-9.3 / -5.0	20.5	C 34 H 28 35Cl O 4
		+6.7 / +3.6	8.5	C 27 H 36 37Cl 3 O 4
		+5.2 / +2.8	7.5	C 26 H 36 35Cl 2 37Cl O 5
		-3.6 / -1.9	12.5	C 29 H 33 37Cl 2 O 5
		-5.2 / -2.8	11.5	C 28 H 33 35Cl 2 O 6
		-14.0 / -7.5	16.5	C 31 H 30 37Cl O 6
		+0.5 / +0.2	3.5	C 23 H 38 35Cl 37Cl 2 O 7
		-1.1 / -0.6	2.5	C 22 H 38 35Cl 3 O 8
		-9.9 / -5.3	7.5	C 25 H 35 35Cl 37Cl O 8
		+14.5 / +7.8	12.5	C 27 H 30 37Cl O 9
		-4.3 / -2.3	-0.5	C 20 H 40 37Cl 3 O 9
		-14.6 / -7.8	3.5	C 22 H 37 37Cl 2 O 10
		-14.1 / -7.5	12.5	C 31 H 35 35Cl 3 Na
		+11.8 / +6.3	18.5	C 34 H 30 37Cl 2 Na
		+10.3 / +5.5	17.5	C 33 H 30 35Cl 2 O Na
		+1.5 / +0.8	22.5	C 36 H 27 37Cl O Na
		-17.3 / -9.3	9.5	C 29 H 37 37Cl 3 O Na
		+15.9 / +8.5	9.5	C 28 H 35 35Cl 37Cl 2 O 2 Na
		+14.4 / +7.7	8.5	C 27 H 35 35Cl 3 O 3 Na
		+5.6 / +3.0	13.5	C 30 H 32 35Cl 37Cl O 3 Na
		-4.8 / -2.5	17.5	C 32 H 29 35Cl O 4 Na
		+11.2 / +6.0	5.5	C 25 H 37 37Cl 3 O 4 Na
		+9.7 / +5.2	4.5	C 24 H 37 35Cl 2 37Cl O 5 Na
		+0.9 / +0.5	9.5	C 27 H 34 37Cl 2 O 5 Na
		-0.7 / -0.4	8.5	C 26 H 34 35Cl 2 O 6 Na
		-9.5 / -5.1	13.5	C 29 H 31 37Cl O 6 Na
		+5.0 / +2.7	0.5	C 21 H 39 35Cl 37Cl 2 O 7 Na
		+3.4 / +1.8	-0.5	C 20 H 39 35Cl 3 O 8 Na
		-5.4 / -2.9	4.5	C 23 H 36 35Cl 37Cl O 8 Na
		-15.7 / -8.4	8.5	C 25 H 33 35Cl O 9 Na
		-10.1 / -5.4	0.5	C 20 H 38 37Cl 2 O 10 Na

Figure S30. HRMS spectrum of Merochlorin I (**3**)

Page: 1

[Elemental Composition]

Data : FAB-Q995
Sample: CNH189 M2-4 T5
Note : m-NBA
Inlet : Direct
RT : 4.16 min

Date : 15-Jun-2018 18:12

Ion Mode : FAB+
Scan#: (181,205)

Elements : C 100/0, H 100/0, Cl 4/2(35Cl 4/0, 37Cl 4/0), O 10/0, Na 1/0
Mass Tolerance : 20ppm, 5mmu if m/z < 250, 10mmu if m/z > 500
Unsaturation (U.S.) : -0.5 - 50.0

Observed m/z	Int%	Err[ppm / mmu]	U.S.	Composition
553.1281	0.6	+4.3 / +2.4	20.5	C 35 H 28 35Cl 3
		-4.2 / -2.3	25.5	C 38 H 25 35Cl 37Cl
		+11.2 / +6.2	13.5	C 31 H 33 37Cl 4
		+9.8 / +5.4	12.5	C 30 H 33 35Cl 2 37Cl 2 O
		+1.2 / +0.7	17.5	C 33 H 30 37Cl 3 O
		+8.3 / +4.6	11.5	C 29 H 33 35Cl 4 O 2
		-0.2 / -0.1	16.5	C 32 H 30 35Cl 2 37Cl O 2
		-8.8 / -4.9	21.5	C 35 H 27 37Cl 2 O 2
		-10.3 / -5.7	20.5	C 34 H 27 35Cl 2 O 3
		+5.2 / +2.9	8.5	C 27 H 35 35Cl 37Cl 3 O 3
		+3.7 / +2.1	7.5	C 26 H 35 35Cl 3 37Cl O 4
		-4.8 / -2.7	12.5	C 29 H 32 35Cl 37Cl 2 O 4
		-6.3 / -3.5	11.5	C 28 H 32 35Cl 3 O 5
		-14.8 / -8.2	16.5	C 31 H 29 35Cl 37Cl O 5
		+0.6 / +0.3	4.5	C 24 H 37 37Cl 4 O 5
		+17.3 / +9.6	16.5	C 30 H 27 35Cl 2 O 6
		-0.9 / -0.5	3.5	C 23 H 37 35Cl 2 37Cl 2 O 6
		-9.4 / -5.2	8.5	C 26 H 34 37Cl 3 O 6
		-2.3 / -1.3	2.5	C 22 H 37 35Cl 4 O 7
		-10.9 / -6.0	7.5	C 25 H 34 35Cl 2 37Cl O 7
		+12.8 / +7.1	12.5	C 27 H 29 35Cl 37Cl O 8
		-5.4 / -3.0	-0.5	C 20 H 39 35Cl 37Cl 3 O 8
		-15.4 / -8.5	3.5	C 22 H 36 35Cl 37Cl 2 O 9
		-16.9 / -9.4	2.5	C 21 H 36 35Cl 3 O 10
		+16.7 / +9.2	3.5	C 21 H 34 35Cl 2 37Cl O 10
		+8.2 / +4.5	8.5	C 24 H 31 37Cl 2 O 10
		+8.7 / +4.8	17.5	C 33 H 29 35Cl 3 Na
		+0.1 / +0.1	22.5	C 36 H 26 35Cl 37Cl Na
		-18.0 / -10.0	9.5	C 29 H 36 35Cl 37Cl 3 Na
		+15.6 / +8.6	10.5	C 29 H 34 37Cl 4 Na
		+14.1 / +7.8	9.5	C 28 H 34 35Cl 2 37Cl 2 O Na
		+5.6 / +3.1	14.5	C 31 H 31 37Cl 3 O Na
		+12.6 / +7.0	8.5	C 27 H 34 35Cl 4 O 2 Na
		+4.1 / +2.3	13.5	C 30 H 31 35Cl 2 37Cl O 2 Na
		-4.4 / -2.5	18.5	C 33 H 28 37Cl 2 O 2 Na
		-5.9 / -3.3	17.5	C 32 H 28 35Cl 2 O 3 Na
		+9.5 / +5.3	5.5	C 25 H 36 35Cl 37Cl 3 O 3 Na
		+8.1 / +4.5	4.5	C 24 H 36 35Cl 3 37Cl O 4 Na
		-0.5 / -0.3	9.5	C 27 H 33 35Cl 37Cl 2 O 4 Na
		-1.9 / -1.1	8.5	C 26 H 33 35Cl 3 O 5 Na
		-10.5 / -5.8	13.5	C 29 H 30 35Cl 37Cl O 5 Na
		+5.0 / +2.7	1.5	C 22 H 38 37Cl 4 O 5 Na
		+3.5 / +1.9	0.5	C 21 H 38 35Cl 2 37Cl 2 O 6 Na
		-5.0 / -2.8	5.5	C 24 H 35 37Cl 3 O 6 Na
		+2.0 / +1.1	-0.5	C 20 H 38 35Cl 4 O 7 Na
		-6.5 / -3.6	4.5	C 23 H 35 35Cl 2 37Cl O 7 Na
		-15.1 / -8.3	9.5	C 26 H 32 37Cl 2 O 7 Na
		-16.5 / -9.1	8.5	C 25 H 32 35Cl 2 O 8 Na
		+17.1 / +9.5	9.5	C 25 H 30 35Cl 37Cl O 8 Na
		-11.1 / -6.1	0.5	C 20 H 37 35Cl 37Cl 2 O 9 Na
		-12.6 / -6.9	-0.5	C 19 H 37 35Cl 3 O 10 Na
		+12.5 / +6.9	5.5	C 22 H 32 37Cl 2 O 10 Na

Figure S31. HRMS spectrum of Merochlorin J (**4**)

[Elemental Composition]   Page: 1

Data : FAB-Q651   Date : 03-Apr-2018 13:20
Sample: CNH189.493-T4
Note : m-NBA
Inlet : Direct   Ion Mode : FAB+
RT : 0.82 min   Scan#: (26,51)
Elements : C 100/0, H 100/0, Cl 2/0(35Cl 2/0, 37Cl 2/0), N 10/0, O 10/0
Mass Tolerance : 20ppm, 5mmu if m/z < 250, 10mmu if m/z > 500
Unsaturation (U.S.) : 5.0 - 20.0

Observed m/z	Int%	Err[ppm / mmu]	U.S.	Composition
494.2311	100.0	-2.1 / -1.0	13.0	C 32 H 38 35Cl 37Cl
		-14.3 / -7.1	12.5	C 31 H 38 35Cl 2 N
		+11.2 / +5.5	13.0	C 30 H 36 35Cl 2 N 2
		+1.6 / +0.8	18.0	C 33 H 33 37Cl N 2
		-10.6 / -5.2	17.5	C 32 H 33 35Cl N 3
		+14.9 / +7.4	18.0	C 31 H 31 35Cl N 4
		-1.8 / -0.9	10.0	C 25 H 36 37Cl 2 N 6
		-14.0 / -6.9	9.5	C 24 H 36 35Cl 37Cl N 7
		+11.5 / +5.7	10.0	C 23 H 34 35Cl 37Cl N 8
		-0.7 / -0.3	9.5	C 22 H 34 35Cl 2 N 9
		-10.2 / -5.1	14.5	C 25 H 31 37Cl N 9
		+15.2 / +7.5	15.0	C 24 H 29 37Cl N 10
		-13.3 / -6.6	17.0	C 34 H 35 35Cl O
		+12.2 / +6.0	17.5	C 33 H 33 35Cl N O
		-4.5 / -2.2	9.5	C 27 H 38 37Cl 2 N 3 O
		-16.7 / -8.2	9.0	C 26 H 38 35Cl 37Cl N 4 O
		+8.8 / +4.3	9.5	C 25 H 36 35Cl 37Cl N 5 O
		-3.4 / -1.7	9.0	C 24 H 36 35Cl 2 N 6 O
		-13.0 / -6.4	14.0	C 27 H 33 37Cl N 6 O
		+12.5 / +6.2	14.5	C 26 H 31 37Cl N 7 O
		+0.3 / +0.1	14.0	C 25 H 31 35Cl N 8 O
		+4.0 / +2.0	19.0	C 26 H 26 N 10 O
		-16.4 / -8.1	6.0	C 19 H 36 37Cl 2 N 10 O
		-7.2 / -3.6	9.0	C 29 H 40 37Cl 2 O 2
		-19.4 / -9.6	8.5	C 28 H 40 35Cl 37Cl N O 2
		+18.2 / +9.0	9.5	C 28 H 38 37Cl 2 N O 2
		+6.1 / +3.0	9.0	C 27 H 38 35Cl 37Cl N 2 O 2
		-6.1 / -3.0	8.5	C 26 H 38 35Cl 2 N 3 O 2
		-15.7 / -7.8	13.5	C 29 H 35 37Cl N 3 O 2
		+19.3 / +9.5	9.0	C 25 H 36 35Cl 2 N 4 O 2
		+9.8 / +4.8	14.0	C 28 H 33 37Cl N 4 O 2
		-2.4 / -1.2	13.5	C 27 H 33 35Cl N 5 O 2
		+1.3 / +0.6	18.5	C 28 H 28 N 7 O 2
		-19.1 / -9.4	5.5	C 21 H 38 37Cl 2 N 7 O 2
		+6.4 / +3.2	6.0	C 20 H 36 37Cl 2 N 8 O 2
		-5.8 / -2.9	5.5	C 19 H 36 35Cl 37Cl N 9 O 2
		-18.0 / -8.9	5.0	C 18 H 36 35Cl 2 N 10 O 2
		+19.6 / +9.7	6.0	C 18 H 34 35Cl 37Cl N 10 O 2
		-8.8 / -4.4	8.0	C 28 H 40 35Cl 2 O 3
		-18.4 / -9.1	13.0	C 31 H 37 37Cl O 3
		+16.6 / +8.2	8.5	C 27 H 38 35Cl 2 N O 3
		+7.0 / +3.5	13.5	C 30 H 35 37Cl N O 3
		-5.1 / -2.5	13.0	C 29 H 35 35Cl N 2 O 3
		-1.4 / -0.7	18.0	C 30 H 30 N 4 O 3
		+3.7 / +1.8	5.5	C 22 H 38 37Cl 2 N 5 O 3
		-8.5 / -4.2	5.0	C 21 H 38 35Cl 37Cl N 6 O 3
		+16.9 / +8.4	5.5	C 20 H 36 35Cl 37Cl N 7 O 3
		+4.7 / +2.3	5.0	C 19 H 36 35Cl 2 N 8 O 3
		-4.8 / -2.4	10.0	C 22 H 33 37Cl N 8 O 3
		-17.0 / -8.4	9.5	C 21 H 33 35Cl N 9 O 3
		+8.4 / +4.2	10.0	C 20 H 31 35Cl N 10 O 3
		+17.6 / +8.7	13.0	C 30 H 35 35Cl O 4
		-4.2 / -2.1	17.5	C 32 H 32 N O 4
		+0.9 / +0.5	5.0	C 24 H 40 37Cl 2 N 2 O 4
		+14.2 / +7.0	5.0	C 22 H 38 35Cl 37Cl N 4 O 4

[Elemental Composition]   Page: 2

Err[ppm / mmu]	U.S.	Composition
-7.5 / -3.7	9.5	C 24 H 35 37Cl N 5 O 4
-19.7 / -9.8	9.0	C 23 H 35 35Cl N 6 O 4
+17.9 / +8.8	10.0	C 23 H 33 37Cl N 6 O 4
+5.7 / +2.8	9.5	C 22 H 33 35Cl N 7 O 4
-16.0 / -7.9	14.0	C 24 H 30 N 8 O 4
+9.4 / +4.7	14.5	C 23 H 28 N 9 O 4
-10.3 / -5.1	9.0	C 26 H 37 37Cl N 2 O 5
+15.2 / +7.5	9.5	C 25 H 35 37Cl N 3 O 5
+3.0 / +1.5	9.0	C 24 H 35 35Cl N 4 O 5
-18.7 / -9.3	13.5	C 26 H 32 N 5 O 5
+6.7 / +3.3	14.0	C 25 H 30 N 6 O 5
+3.3 / +1.6	6.0	C 17 H 33 37Cl N 10 O 5
+12.5 / +6.2	9.0	C 27 H 37 37Cl O 6
+0.3 / +0.1	8.5	C 26 H 37 35Cl N O 6
+4.0 / +2.0	13.5	C 27 H 32 N 3 O 6
+0.6 / +0.3	5.5	C 19 H 35 37Cl N 7 O 6
-11.6 / -5.7	5.0	C 18 H 35 35Cl N 8 O 6
+13.9 / +6.8	5.5	C 17 H 33 35Cl N 9 O 6
-7.9 / -3.9	10.0	C 19 H 30 N 10 O 6
+1.3 / +0.6	13.0	C 29 H 34 O 7
-2.1 / -1.0	5.0	C 21 H 37 37Cl N 4 O 7
+11.1 / +5.5	5.0	C 19 H 35 35Cl N 6 O 7
-10.6 / -5.2	9.5	C 21 H 32 N 7 O 7
+14.8 / +7.3	10.0	C 20 H 30 N 8 O 7
-13.3 / -6.6	9.0	C 23 H 34 N 4 O 8
+12.1 / +6.0	9.5	C 22 H 32 N 5 O 8
-16.0 / -7.9	8.5	C 25 H 36 N O 9
+9.4 / +4.6	9.0	C 24 H 34 N 2 O 9
-2.5 / -1.2	5.5	C 16 H 32 N 9 O 9
-5.2 / -2.6	5.0	C 18 H 34 N 6 O 10

Figure S32. HRMS spectrum of Compound **10**

[Mass Spectrum]
Data : EI-A768   Date : 25-May-2021 14:53
RT : 1.03 min   Scan# : (51,60)
Elements : C 100/0, H 100/0, O 10/0
Mass Tolerance : 10ppm, 5mmu if m/z < 500, 10mmu if m/z > 1000
Unsaturation (U.S.) : -0.5 - 20.0

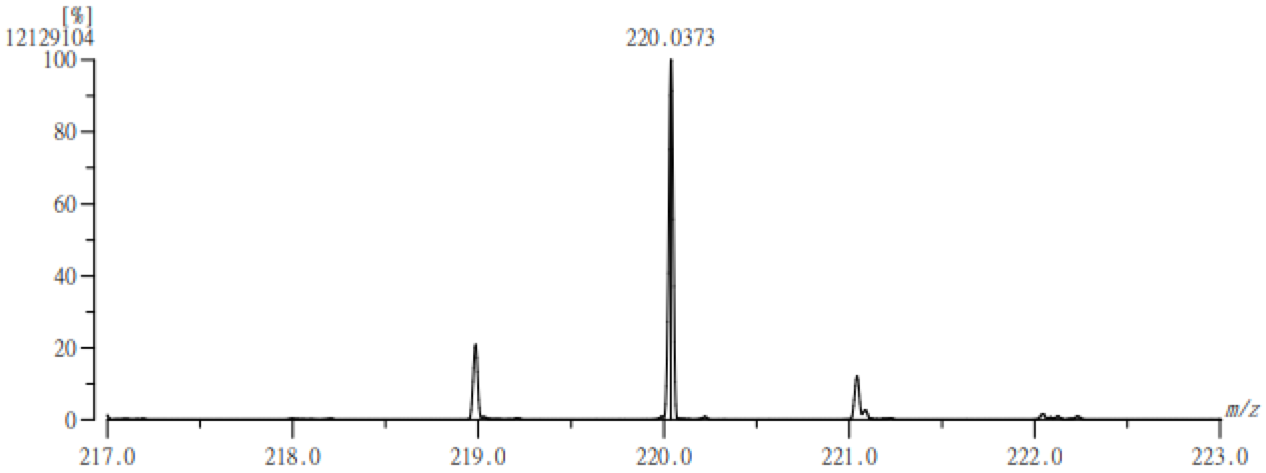

	Observed m/z	Int%	Err[ppm / mmu]	U.S.	Composition
1	220.0373	100.00	+0.6 / +0.1	8.0	C11 H8 O5

Figure S33. Simulated conformer models of two possible diastereomers of **1** (18*R*, 18*S*)

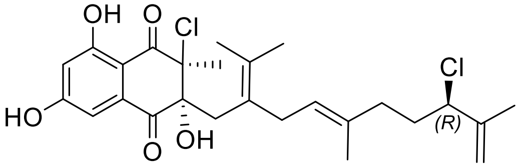

Diastereomer 1 (18*R*)

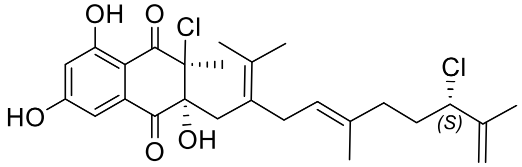

Diastereomer 2 (18*S*)

Figure S34. Simulated conformer models of four possible diastereomers of **4** (15*R*/18*R*, 15*R*/18*S*, 15*S*/18*R*, and 15*S*/18*S*)

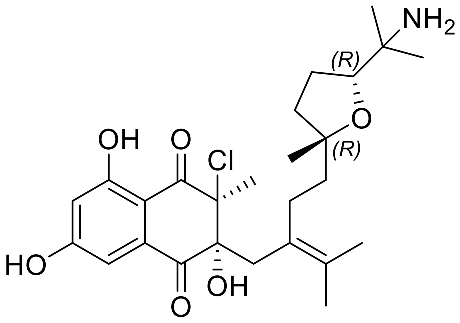

Diastereomer 1 (15*R*/18*R*)

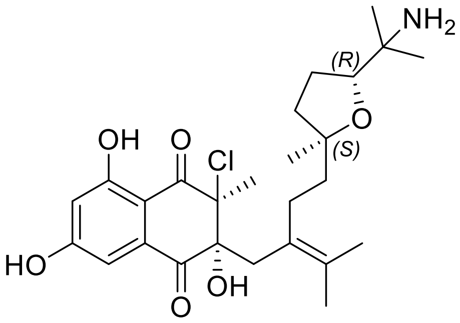

Diastereomer 2 (15*R*/18*S*)

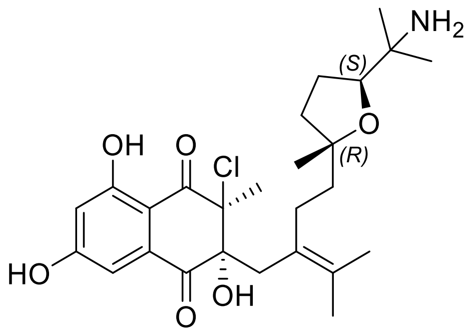

Diastereomer 3 (15*S*/18*R*)

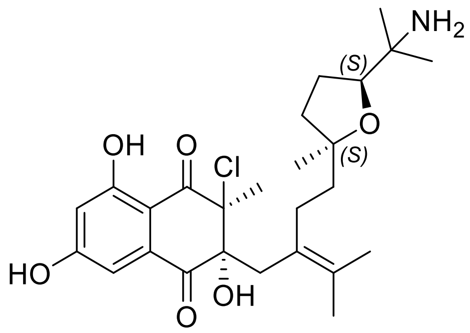

Diastereomer 4 (15*S*/18*S*)

Table S1. Experimental (Exp.) and calculated (Cal.) chemical shift values (CS, δ) of merochlorin G (**1**)

Number	Atom	calculated 18*R*	calculated 18*S*	experimental
1	c	108.16	107.97	108.9
2	c	159.57	159.78	162.9
3	c	108.16	108.45	107.4
4	c	135.65	135.77	134.7
5	c	112.43	112.4	109.9
6	c	163.43	163.42	165.3
9	c	203.67	203.5	195.1
10	c	89.71	89.48	84
11	c	86.76	86.36	74.5
12	c	200.54	201.1	194.7
16	c	47.96	47.54	38.6
17	c	132.19	132.08	131.6
18	c	38.26	38.4	31.3
19	c	130.26	130.52	123.5
20	c	137.59	138.27	134
22	c	137.66	138.47	125.1
23	c	25.3	25.26	20.6
24	c	25.52	25.6	20.9
25	c	42.54	43.81	36.6
26	c	20.92	20.27	18.1
27	c	38.71	39.35	34.8
28	c	65.66	66.75	66.4
29	c	148.71	149.75	144.3

30	c	26.11	25.65	17
32	c	116.44	116.23	114.2
33	c	31.86	31.99	15.9

Table S2. Experimental (Exp.) and calculated (Cal.) chemical shift values (CS, δ) of merochlorin J (**4**)

Number	Atom	15*R*/18*R*	15*R*/18*S*	15*S*/18*R*	15*S*/18*S*	experimental
1	c	112.9	113.67	111.18	113.16	107.7
2	c	165.02	163.53	165.74	165.26	164.1
3	c	110.42	113.85	108.91	109.53	107.2
4	c	135.27	135.75	135.7	135.54	134.9
5	c	110.41	111.33	109.25	110.05	107.8
6	c	166.57	163.95	166.75	166.66	165.2
7	c	203.08	203.22	203.88	202.65	195.3
8	c	88.89	89.23	88.6	89.18	84
9	c	91.95	87.46	92.44	91.74	76.1
10	c	197.14	199.27	197.43	197.46	193.6
15	c	35.16	33.01	33.85	34.79	18.1
17	c	48.89	48.77	44.52	48.75	37.9
18	c	130.88	132.69	131.53	130.58	126.4
20	c	140.95	139.64	140.77	140.89	129.2
21	c	35.26	34.4	33.55	35.31	26.3
22	c	26.89	25.88	26.16	27.14	20

23	c	28.69	27.65	27.08	28.8	20
24	c	45.84	45.39	47.81	46.01	39.7
25	c	85.48	84.72	86.23	85.87	83.4
27	c	88.78	88.85	87.2	88.08	81
28	c	33.45	31.99	33.48	31.36	25.9
29	c	40.48	38.69	47.77	42.57	36.3
30	c	34.35	35.84	33.14	31.59	24.7
31	c	56.02	56.17	56.78	56.55	55.5
32	c	35.05	32.38	31.62	36.4	19.7
33	c	30.62	31.58	31.68	29.91	22.8
35	h	5.98	5.95	6.05	6.12	6.62
36	h	6.42	6.54	6.21	6.12	6.81
39	h	0.76	0.73	0.77	0.82	1.81
40	h	1.39	1.25	1.45	1.35	1.81
41	h	2.33	2.13	2.29	2.29	1.81
42	h	3.22	3.24	3.28	3.32	2.45
43	h	1.9	1.59	1.69	1.51	2.31
44	h	2.99	2.6	2.98	3.04	6.16
45	h	1.25	0.99	1.34	0.9	2.23
46	h	1.71	1.56	1.98	2.04	1.65
47	h	1.35	1.43	1.45	1.65	1.05
48	h	1.6	1.26	1.17	1.4	1.05
49	h	1.43	1.11	1.7	1.49	1.05
50	h	2.02	2.18	2.54	1.47	1.47

51	h	1.32	1.09	1.21	1.76	1.47
52	h	1.67	1.28	1.28	1.91	1.47
53	h	1.12	0.93	0.94	1.33	1.42
54	h	1.31	1.07	0.96	0.89	1.17
55	h	3.4	3.69	3.62	3.53	3.98
56	h	1.55	1.38	1.32	1.65	1.88
57	h	1.36	1.25	1.35	1.4	1.64
58	h	1.33	1.11	1.54	1.19	1.56
59	h	1.73	1.67	1.4	0.96	1.56
60	h	0.86	0.77	0.62	0.64	1.06
61	h	1.3	0.87	1.01	1.26	1.06
62	h	0.8	0.5	1.33	1.03	1.06
63	h	1.22	0.71	0.87	0.99	1.14
64	h	0.99	1.08	1.48	1.28	1.14
65	h	0.92	0.63	0.82	0.99	1.14
66	h	1.15	0.34	0.6	1.06	1.16
67	h	1.31	1.26	1.41	1.04	1.16
68	h	0.95	0.83	1.05	0.72	1.16
